# Associations of Insulin Resistance Surrogate Indices With Component‐Defined Metabolically Unhealthy Phenotype Among Women With Overweight or Obesity: A Cross‐Sectional Analysis of CHNS and NHANES

**DOI:** 10.1155/ije/5660424

**Published:** 2026-10-14

**Authors:** Xiaoye Zhu, Jinghui Zou, Hong Zhu, Honghong Wang, Yisheng Zhang, Yanfei Jin

**Affiliations:** ^1^ School of Nursing Nanjing Medical University 101 Longmian Road Nanjing 211166 Jiangsu China njmu.edu.cn; ^2^ Department of Obstetrics The Affiliated Lihuili Hospital of Ningbo University 1111 Jiangnan Road Ningbo 315100 Zhejiang China nbu.edu.cn

**Keywords:** cardiometabolic risk, cross-sectional study, HOMA-IR, metabolically unhealthy phenotype, METS-IR, obesity, overweight, triglyceride–glucose index, women

## Abstract

**Background:**

Women with overweight or obesity are metabolically heterogeneous, and body mass index (BMI) alone does not fully characterise their endocrine and metabolic health. We examined associations between five insulin resistance surrogate indices and a component‐defined metabolically unhealthy phenotype (MUP) to characterise insulin resistance–related metabolic variation in this population.

**Methods:**

This cross‐sectional analysis used the China Health and Nutrition Survey (CHNS) 2009 for discovery and the National Health and Nutrition Examination Survey (NHANES) 2011–2018 for independent replication. Final samples comprised 1751 CHNS and 2953 NHANES participants. The indices were homeostasis model assessment of insulin resistance (HOMA‐IR), triglyceride–glucose index (TyG), triglyceride‐to‐high‐density lipoprotein cholesterol ratio (TG/HDL‐C), triglyceride–glucose body mass index (TyG‐BMI) and metabolic score for insulin resistance (METS‐IR). MUP was defined as at least two abnormal metabolic components. Modified Poisson regression estimated prevalence ratios (PRs) per 1‐standard‐deviation increment and across quartiles. Fully adjusted models included 1721 CHNS and 2799 NHANES participants. Receiver operating characteristic curves and areas under the curve (AUCs) provided a descriptive, exploratory assessment of internal discrimination.

**Results:**

All five indices were positively associated with MUP in both datasets. Fully adjusted PRs per 1‐standard‐deviation increment ranged from 1.027 (95% confidence interval 1.011–1.043) for HOMA‐IR to 1.133 (1.110–1.157) for TyG in CHNS and from 1.058 (1.024–1.094) for TG/HDL‐C to 1.168 (1.144–1.192) for TyG in NHANES. Positive quartile gradients were observed for all indices (*p* for trend < 0.001). Exploratory internal discrimination for MUP showed different patterns across the two datasets.

**Conclusions:**

Higher values of all five indices were associated with MUP among women with overweight or obesity in both datasets. These findings highlight the co‐occurrence of insulin resistance–related glucose and lipid disturbances with clustered metabolic abnormalities. They support considering metabolic function alongside adiposity when characterising endocrine‐metabolic heterogeneity among women with overweight or obesity.

## 1. Background

Women with overweight or obesity do not constitute a metabolically uniform population. Within the same body mass index (BMI) category, lipid abnormalities, central adiposity, dysglycaemia and blood pressure elevation may cluster to varying degrees, giving rise to metabolically healthy and metabolically unhealthy phenotypes (MUPs) [1, 2]. BMI alone also provides limited information on body‐fat distribution and individual‐level cardiometabolic impairment, supporting the need to consider metabolic health in addition to adiposity status [3]. Characterising metabolic heterogeneity among women with excess adiposity is therefore relevant to women’s metabolic health and population‐level assessment of obesity‐related cardiometabolic risk.

Lipid dysregulation is a key feature of obesity‐related metabolic risk. Raised triglycerides (TG), reduced high‐density lipoprotein cholesterol (HDL‐C), dysglycaemia, elevated blood pressure and central adiposity are recognised components of clustered cardiometabolic risk [4]. Differences in insulin sensitivity, fat distribution and adipose tissue function contribute to the biological heterogeneity of obesity, providing a rationale for examining insulin resistance–related measures alongside metabolic phenotype [1]. The homeostasis model assessment of insulin resistance (HOMA‐IR) is widely used as an insulin‐dependent reference index of insulin resistance, although it requires fasting insulin measurement [5]. By contrast, several noninsulin surrogate indices can be derived from routine lipid, glucose and anthropometric measurements.

These indices should not be treated as interchangeable markers. The triglyceride–glucose index (TyG) reflects triglyceride–glucose coupling and has been evaluated as a noninsulin surrogate of insulin resistance, including in comparison with the euglycaemic‐hyperinsulinaemic clamp [6, 7]. The triglyceride‐to‐high‐density lipoprotein cholesterol ratio (TG/HDL‐C) represents a lipid ratio dimension related to atherogenic dyslipidaemia and insulin resistance, with performance that may differ across populations [8, 9]. The triglyceride–glucose body mass index (TyG‐BMI) integrates triglyceride–glucose information with general adiposity and has been proposed as a practical surrogate marker for insulin resistance [10]. The metabolic score for insulin resistance (METS‐IR) incorporates glucose, TG, HDL‐C and BMI as a composite metabolic insulin resistance index [11]. The metabolic information incorporated in each index may help clarify how insulin resistance–related measures relate to the clustering of metabolic abnormalities among women with overweight or obesity.

Evidence remains limited on whether associations between these indices and component‐defined MUP are consistent across populations of adult women with excess adiposity. Findings from a single dataset may be influenced by population structure, BMI distribution, ethnicity, survey design and laboratory procedures. CHNS and NHANES differ in study design, source population, laboratory procedures and weighting requirements [12, 13] and NHANES analyses require consideration of complex survey design and subsample weights [14, 15]. Accordingly, separate analyses of the two datasets allow consistency across CHNS and NHANES to be assessed while preserving database‐specific definitions and survey designs.

In this study, we evaluated associations of HOMA‐IR, TyG, TyG‐BMI, TG/HDL‐C and METS‐IR with component‐defined MUP among adult women with overweight or obesity, with an emphasis on their relevance to endocrine‐metabolic heterogeneity in this population. CHNS 2009 served as the discovery dataset and NHANES 2011–2018 as an independent replication dataset. Continuous and quartile‐based associations, component‐specific outcomes, composite metabolic dysregulation burden and consistency between CHNS and NHANES were examined to describe the relationships between these indices and metabolic phenotype across the two datasets, alongside an exploratory assessment of internal discrimination. Component‐specific analyses distinguished direct formula‐to‐outcome overlap from associations with metabolic components that did not enter the index formulas.

## 2. Methods

### 2.1. Study Design and Data Sources

This cross‐sectional analysis used CHNS 2009 as the discovery dataset and NHANES 2011–2018 as an independent replication dataset. The datasets were analysed separately without pooling individual‐level records across databases. We evaluated associations of lipid‐related and insulin resistance surrogate indices derived from fasting biochemical and anthropometric measurements with component‐defined MUP and assessed their exploratory internal discrimination among adult women with overweight or obesity.

For CHNS, the analysis was based on the 2009 biomarker survey, which included fasting blood biomarkers, anthropometry and blood pressure measurements [16]. The initial CHNS biomarker sample included 9549 participants. After linkage with sex and birth‐year information, the sample was restricted to women aged 18 years or older with available height and weight, BMI ≥ 24 kg/m^2^, available fasting glucose, fasting insulin, triglyceride and HDL‐C measurements and available waist circumference (WC) and blood pressure measurements. Known pregnant women were excluded, while participants with missing pregnancy status were retained as unknown. The final CHNS analytic sample included 1751 adult women with overweight or obesity.

For NHANES, data from the 2011–2018 cycles were combined. The initial merged sample included 39,156 participants. The sample was restricted to women aged 20 years or older who were not pregnant, had BMI ≥ 25 kg/m^2^, were included in the fasting subsample with available 8‐year fasting subsample weights and had available fasting glucose, fasting insulin, triglyceride, HDL‐C, WC and blood pressure measurements. The final NHANES analytic sample included 2953 adult women with overweight or obesity. NHANES analyses accounted for fasting subsample weights, strata and primary sampling units (PSUs).

### 2.2. Assessment of Lipid‐Related and Insulin Resistance Surrogate Indices

Five surrogate indices were evaluated and grouped according to their constituent metabolic measures. HOMA‐IR was treated as the insulin‐dependent reference index; TyG as a triglyceride–glucose coupling index; TG/HDL‐C as a lipid ratio index; TyG‐BMI as an adiposity‐integrated TyG; and METS‐IR as a composite metabolic insulin resistance index. The indices were calculated as follows:

 HOMA‐IR = fasting insulin (μU/mL) × fasting glucose (mmol/L)/22.5.
 TyG = ln [fasting triglycerides (mg/dL) × fasting glucose (mg/dL)/2].
 TG/HDL‐C = triglycerides/HDL‐C, using the same concentration unit.
 TyG‐BMI = TyG × BMI.
 METS‐IR = ln [2 × fasting glucose (mg/dL) + fasting triglycerides (mg/dL)] × BMI/ln [HDL‐C (mg/dL)]

Glucose, triglyceride and HDL‐C concentrations were converted to the units specified in each formula before index calculation; ln denotes the natural logarithm.

### 2.3. Definition of MUP

The primary outcome was component‐defined MUP, defined as the presence of at least two of five abnormal metabolic components. Blood pressure abnormality was defined as systolic blood pressure ≥ 130 mmHg, diastolic blood pressure ≥ 85 mmHg, or available database‐specific hypertension history or antihypertensive medication information. Dysglycaemia was defined as fasting glucose ≥ 5.6 mmol/L, HbA1c ≥ 5.7%, or available database‐specific diabetes history or medication information. Elevated TG were defined as ≥ 1.7 mmol/L, reduced HDL‐C as < 1.29 mmol/L and elevated WC as ≥ 80 cm in CHNS and ≥ 88 cm in NHANES. Detailed database‐specific operational definitions, reflecting differences in available questionnaire and medication variables, are provided in Supporting Table S1. An alternative waist‐circumference cut‐off phenotype was examined as a sensitivity outcome. The ≥ 2‐component threshold is an investigator‐defined composite based on harmonised metabolic‐syndrome components rather than a validated clinical diagnostic criterion, and the resulting classification of women as metabolically unhealthy is therefore study‐specific rather than a formal clinical diagnosis.

Additional sensitivity analyses included MUP defined as at least three abnormal metabolic components, the common 85‐cm waist‐circumference sensitivity phenotype and analyses restricted to women with obesity. High HOMA‐IR was defined as values in the database‐specific highest quartile and was evaluated as a sensitivity outcome in exploratory ROC analyses; HOMA‐IR was excluded from the predictor set for this outcome.

### 2.4. Covariates

Age and total energy intake were modelled as continuous variables and standardised within each database. In CHNS, residence was classified as urban or rural; education as no schooling, primary school, middle school, high school or technical school, or college or above; and alcohol consumption as no or yes. In NHANES, race/ethnicity was classified as Mexican American, other Hispanic, non‐Hispanic White, non‐Hispanic Black, non‐Hispanic Asian, or other race/multiracial; education as less than 9th grade, 9th–11th grade, high school or GED, some college or associate degree, or college graduate or above; and smoking as never, former, or current. The primary fully adjusted model applied the same covariate set, excluding BMI, identically to all five indices, so that the resulting estimates were directly comparable. Fully adjusted models additionally including BMI for HOMA‐IR, TyG and TG/HDL‐C are examined separately as a secondary sensitivity check. BMI is not, and cannot be, added as an additional covariate in the TyG‐BMI and METS‐IR models in either specification because it is a component of both indices; these BMI‐adjusted estimates for HOMA‐IR, TyG and TG/HDL‐C are reported only as secondary sequential‐model results (Supporting Figure S1) and are not compared against TyG‐BMI or METS‐IR.

### 2.5. Statistical Analysis

Baseline characteristics were described according to metabolic phenotype. Continuous variables were presented as mean ± standard deviation (SD) or median [interquartile range], according to their distributions. CHNS group comparisons used Welch two‐sample *t*‐tests, Wilcoxon rank‐sum tests or Pearson chi‐square tests, as appropriate. NHANES estimates and group comparisons accounted for fasting subsample weights, strata and PSUs and used survey‐weighted *t*‐tests, survey‐weighted Wilcoxon rank tests or Rao–Scott chi‐square tests, as appropriate [14].

Empirical Spearman correlation coefficients were calculated between HOMA‐IR and each noninsulin surrogate index within the final analytic sample of each database. Each index was standardised within its respective database, and database‐specific SDs and index distributions are provided in Supporting Table S2. Associations with component‐defined MUP were estimated separately for each index using modified Poisson regression with a log link to obtain prevalence ratios (PRs) and 95% confidence intervals (CIs) [17]. CHNS models used HC0 standard errors clustered by community identifier (COMMID; 217 communities). NHANES models used survey‐weighted quasipoisson regression incorporating the 8‐year fasting subsample weight (WTSAF8YR, calculated for each 2011–2018 cycle as the corresponding 2‐year fasting weight WTSAF2YR divided by 4), the stratum variable SDMVSTRA and the primary‐sampling‐unit variable SDMVPSU (R survey package, nest = TRUE, Taylor‐series linearised variance estimation). Database‐specific empirical quartiles were defined from the unweighted index distributions, with Q1 as the reference group. Tests for trend modelled the ordered quartile number as a continuous term.

Four sequential adjustment models were used. Model 1 was unadjusted. In CHNS, Model 2 included age, urban or rural residence and education, and Model 3 additionally included alcohol consumption and total energy intake. In NHANES, Model 2 included age, race/ethnicity and education, and Model 3 additionally included smoking and total energy intake. Model 3, common to all five indices and excluding BMI, was treated as the primary fully adjusted comparison across indices. Model 4 additionally included BMI for HOMA‐IR, TyG and TG/HDL‐C; BMI was not added to the TyG‐BMI or METS‐IR models because it is a component of both indices; Model 4 was retained only as a secondary sequential sensitivity check for HOMA‐IR, TyG and TG/HDL‐C (Supporting Figure S1) and was not used to rank these indices against TyG‐BMI or METS‐IR. No missing values were imputed. Fully adjusted (Model 3) analyses used complete cases and included 1721 CHNS participants and 2799 NHANES participants.

Exploratory internal discrimination was assessed using empirical receiver operating characteristic (ROC) curves and areas under the curve (AUCs), with 95% CIs estimated using the DeLong method. Within each database, paired DeLong tests compared the AUC of each nonreference index with that of HOMA‐IR [18]. ROC analyses were unweighted empirical analyses; NHANES fasting subsample weights, strata and PSUs were not incorporated. Cut‐off values were identified using the Youden index and were interpreted as internally derived exploratory thresholds rather than universal clinical cut‐offs [19]. All analyses were performed using R Version 4.6.0, and two‐sided *p* values < 0.05 were considered statistically significant.

A survey‐design‐aware companion ROC analysis was also conducted for NHANES. Survey‐weighted AUCs were estimated using the WeightedROC package with WTSAF8YR as the observation weight, and 95% confidence intervals were obtained from a stratified, cluster‐based bootstrap (500 resamples drawing PSUs with replacement within SDMVSTRA strata). This weighted analysis is reported separately from the primary unweighted empirical ROC analysis, and a survey‐weighted paired DeLong test was not implemented.

### 2.6. Composite Metabolic Dysregulation Burden and Consistency Analysis

To complement the binary component‐defined phenotype, a continuous metabolic dysregulation burden score was constructed from five adverse‐direction metabolic domains. Adiposity was represented by standardised WC; blood pressure by the mean of standardised systolic and diastolic blood pressure; glycaemic status by the mean of standardised fasting glucose and HbA1c; TG by standardised triglyceride concentration; and HDL‐C by the negative of standardised HDL‐C. All variables were standardised within each database. The primary burden score was calculated as the arithmetic mean of the five domain scores, with higher values indicating a more adverse metabolic profile. Categorical history and medication variables used in the binary MUP definition were not incorporated into the continuous burden score. The burden score was treated as a complementary continuous representation of the same metabolic domains rather than an independent validation outcome.

Index–burden alignment was assessed using unweighted Spearman and Pearson correlations between each surrogate index and the burden score. Adjusted linear regression models estimated the change in burden score per 1‐SD higher index. CHNS models used HC0 standard errors clustered by community identifier, whereas NHANES models incorporated fasting subsample weights, strata and PSUs. Covariate adjustment used the same common, BMI‐free covariate specification, applied identically to all five indices, that served as the primary comparison for the main association analyses. BMI‐adjusted burden‐score coefficients for HOMA‐IR, TyG and TG/HDL‐C were not used for cross‐index ranking and are not compared against TyG‐BMI or METS‐IR, because BMI is embedded in the latter two index formulas. Unweighted mean burden scores were also summarised across database‐specific index quartiles, and differences between Q4 and Q1 described the quartile gradient. Consistency across CHNS and NHANES was assessed by ranking each index within each database according to its Spearman correlation with the burden score, adjusted beta for the burden score and exploratory AUC for component‐defined MUP. Rank consistency was classified as high when directions agreed and the maximum rank difference between databases was ≤ 1; moderate when directions agreed and the maximum difference was 2; and low when directions differed or the maximum difference exceeded 2.

### 2.7. Sensitivity and Supportive Subgroup Analyses

Sensitivity analyses assessed the consistency of the associations across alternative phenotype definitions and analytic populations. These included MUP defined as at least three abnormal metabolic components, a sensitivity phenotype using a common waist‐circumference cut‐off of ≥ 85 cm in both databases and analyses restricted to women with obesity. To examine formula‐to‐outcome component overlap, separate modified Poisson models evaluated each of the five metabolic components using the same fully adjusted covariate structure as the main analyses. Blood pressure abnormality was examined as a conservative component outcome because blood pressure did not directly enter any of the surrogate‐index formulas.

Supportive subgroup analyses were conducted by age group and BMI category to examine heterogeneity across age and adiposity strata. Age groups were defined as < 45 years, 45–54 years and ≥ 55 years. BMI analyses included an obesity‐only restriction within the primary overweight or obesity population and full BMI‐spectrum analyses after excluding women with underweight. In CHNS, BMI categories were defined as normal weight, 18.5–23.9 kg/m^2^; overweight, 24.0–27.9 kg/m^2^; and obesity, ≥ 28.0 kg/m^2^. In NHANES, the corresponding categories were normal weight, 18.5–24.9 kg/m^2^; overweight, 25.0–29.9 kg/m^2^; and obesity, ≥ 30.0 kg/m^2^. Multiplicative interaction terms between each index and the corresponding subgroup variable were used to assess heterogeneity. These analyses were treated as supportive and are reported in the Supporting Materials.

## 3. Results

### 3.1. Participant Selection and Characteristics

The final analytic samples comprised 1751 adult women with overweight or obesity from CHNS 2009 and 2953 adult women with overweight or obesity from NHANES 2011–2018. The participant selection process is shown in Figure 1. Based on unweighted participant counts, 250 women were classified as metabolically healthy and 1501 as metabolically unhealthy in CHNS; the corresponding numbers in NHANES were 475 and 2478 (unweighted prevalence 83.9%). Incorporating the NHANES fasting‐subsample survey design, the weighted prevalence of the component‐defined MUP was 81.6% (95% CI, 79.6%–83.5%).

**FIGURE 1:**
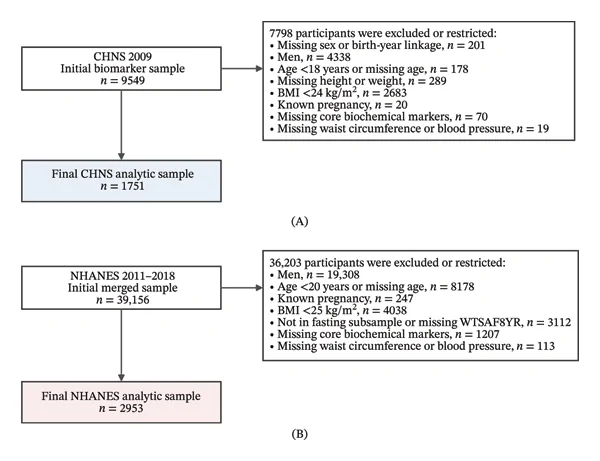
Flowchart of participant selection in CHNS and NHANES. (A) Participant selection in the China Health and Nutrition Survey (CHNS), and (B) participant selection in the National Health and Nutrition Examination Survey (NHANES). The final analytic samples included 1751 CHNS participants and 2953 NHANES participants. CHNS, China Health and Nutrition Survey; NHANES, National Health and Nutrition Examination Survey; BMI, body mass index.

In both datasets, women with MUP were older and had higher BMI, WC, systolic and diastolic blood pressure, fasting glucose, glycated haemoglobin, fasting insulin, TG and surrogate‐index values, together with lower HDL‐C, than metabolically healthy women. Total energy intake did not differ materially between phenotype groups in either dataset. Detailed participant characteristics are presented in Table 1 and Supporting Figure S6.

**TABLE 1:** Baseline characteristics according to metabolic phenotype in CHNS and NHANES.

Characteristic	CHNS			NHANES		
	Metabolically healthy	Metabolically unhealthy	*p* value	Metabolically healthy	Metabolically unhealthy	*p* value
Participants, unweighted n	250	1501	—	475	2478	—
Age, years	45.20 (11.32)	54.41 (12.50)	< 0.001	39.95 (14.51)	51.93 (15.99)	< 0.001
Total energy intake, kcal/day	1955.76 [1621.16, 2334.56]	1943.33 [1577.19, 2291.48]	0.303	1777.00 [1331.00, 2331.00]	1781.00 [1331.00, 2250.00]	0.424
BMI, kg/m^2^	25.79 (1.58)	27.05 (2.46)	< 0.001	29.75 (4.46)	33.82 (6.97)	< 0.001
Waist circumference, cm	82.08 (6.84)	90.25 (7.88)	< 0.001	96.12 (11.61)	108.15 (14.41)	< 0.001
Systolic blood pressure, mmHg	116.73 (12.27)	132.30 (21.02)	< 0.001	111.41 (10.50)	125.66 (17.54)	< 0.001
Diastolic blood pressure, mmHg	76.05 (8.13)	84.07 (11.41)	< 0.001	66.83 (8.64)	70.66 (11.98)	< 0.001
Fasting glucose, mmol/L	4.86 (0.61)	5.69 (1.60)	< 0.001	5.11 (0.41)	6.27 (1.94)	< 0.001
HbA1c, %	5.26 (0.43)	5.91 (0.95)	< 0.001	5.22 (0.30)	5.89 (1.08)	< 0.001
Fasting insulin, μU/mL	9.93 [7.46, 13.74]	13.00 [9.07, 18.90]	< 0.001	7.87 [5.55, 10.72]	13.01 [8.67, 19.24]	< 0.001
Triglycerides, mmol/L	0.99 [0.72, 1.29]	1.67 [1.15, 2.47]	< 0.001	0.81 [0.61, 1.13]	1.25 [0.87, 1.79]	< 0.001
HDL‐C, mmol/L	1.57 (0.30)	1.35 (0.43)	< 0.001	1.66 (0.36)	1.39 (0.40)	< 0.001
HOMA‐IR	2.12 [1.60, 2.95]	3.13 [2.06, 4.87]	< 0.001	1.74 [1.26, 2.47]	3.47 [2.24, 5.32]	< 0.001
TyG	8.22 (0.43)	8.92 (0.68)	< 0.001	8.08 (0.44)	8.71 (0.64)	< 0.001
TyG‐BMI	209.82 [200.48, 220.69]	236.45 [220.28, 258.60]	< 0.001	231.78 [211.83, 265.45]	279.69 [245.38, 330.67]	< 0.001
TG/HDL‐C	0.64 [0.44, 0.90]	1.26 [0.81, 2.02]	< 0.001	0.49 [0.36, 0.69]	0.93 [0.59, 1.43]	< 0.001
METS‐IR	34.67 [33.22, 36.95]	39.87 [36.84, 44.10]	< 0.001	38.81 [35.29, 43.54]	47.66 [41.39, 57.15]	< 0.001

*Note:* Values are mean (SD), median [Q1, Q3], or n as appropriate. CHNS descriptive statistics are unweighted; NHANES descriptive statistics used fasting subsample survey design when applicable. The preferred CHNS population excluded known pregnancy while retaining missing pregnancy status as unknown. HOMA‐IR, homeostasis model assessment of insulin resistance; METS‐IR, metabolic score for insulin resistance; TG/HDL‐C, triglyceride‐to‐high‐density lipoprotein cholesterol ratio; TyG, triglyceride–glucose index; TyG‐BMI, triglyceride–glucose body mass index.

Abbreviations: BMI, body mass index; CHNS, China Health and Nutrition Survey; HDL‐C, high‐density lipoprotein cholesterol; NHANES, National Health and Nutrition Examination Survey.

### 3.2. Main Associations and Quartile‐Based Gradients

The fully adjusted analyses included 1721 CHNS participants and 2799 NHANES participants with complete covariate data. All five indices were positively associated with component‐defined MUP in both datasets. In CHNS, the PR per 1‐SD increment ranged from 1.027 (95% CI, 1.011–1.043) for HOMA‐IR to 1.133 (95% CI, 1.110–1.157) for TyG. In NHANES, the corresponding PRs ranged from 1.058 (95% CI, 1.024–1.094) for TG/HDL‐C to 1.168 (95% CI, 1.144–1.192) for TyG (Table 2; Figure 2).

**TABLE 2:** Associations of lipid‐related and insulin resistance surrogate indices with component‐defined metabolically unhealthy phenotype.

Index	CHNS				NHANES			
	Per 1‐SD PR (95% CI)	*p* value	Q4 vs. Q1 PR (95% CI)	*P* for trend	Per 1‐SD PR (95% CI)	*p* value	Q4 vs. Q1 PR (95% CI)	*P* for trend
HOMA‐IR	1.027 (1.011, 1.043)	< 0.001	1.234 (1.165, 1.308)	< 0.001	1.081 (1.054, 1.109)	< 0.001	1.618 (1.486, 1.762)	< 0.001
TyG	1.133 (1.110, 1.157)	< 0.001	1.418 (1.322, 1.521)	< 0.001	1.168 (1.144, 1.192)	< 0.001	1.599 (1.481, 1.727)	< 0.001
TG/HDL‐C	1.070 (1.047, 1.093)	< 0.001	1.458 (1.359, 1.566)	< 0.001	1.058 (1.024, 1.094)	0.001	1.604 (1.471, 1.749)	< 0.001
TyG‐BMI	1.123 (1.102, 1.144)	< 0.001	1.478 (1.373, 1.590)	< 0.001	1.150 (1.129, 1.172)	< 0.001	1.564 (1.451, 1.686)	< 0.001
METS‐IR	1.131 (1.110, 1.153)	< 0.001	1.537 (1.423, 1.659)	< 0.001	1.163 (1.140, 1.187)	< 0.001	1.648 (1.530, 1.776)	< 0.001

*Note:* Values are prevalence ratios (95% CIs). Per 1‐SD estimates and quartile analyses were derived from the common, fully adjusted, BMI‐free covariate specification applied identically to all five indices, so that estimates share a common adjustment set and are directly comparable across indices. The common model included age, urban/rural residence, education, alcohol drinking and total energy intake (CHNS) or age, race/ethnicity, education, smoking and total energy intake (NHANES). Q1 was the reference group in quartile analyses. BMI‐adjusted estimates for HOMA‐IR, TyG and TG/HDL‐C are reported separately as secondary sequential‐model results in Supporting Figure S1 and are not compared against TyG‐BMI or METS‐IR, because BMI is embedded in the latter two index formulas. HOMA‐IR, homeostasis model assessment of insulin resistance; METS‐IR, metabolic score for insulin resistance; Q, quartile; TG/HDL‐C, triglyceride‐to‐high‐density lipoprotein cholesterol ratio; TyG, triglyceride–glucose index; TyG‐BMI, triglyceride–glucose body mass index.

Abbreviations: BMI, body mass index; CHNS, China Health and Nutrition Survey; CI, confidence interval; NHANES, National Health and Nutrition Examination Survey; PR, prevalence ratio.

**FIGURE 2:**
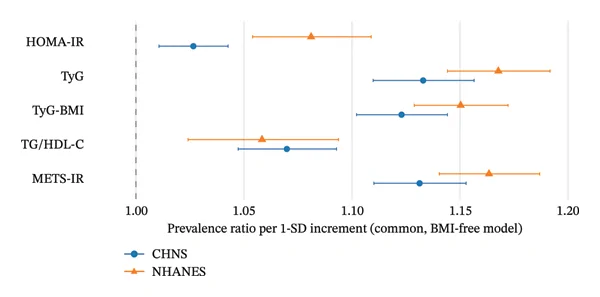
Associations of surrogate indices with component‐defined metabolically unhealthy phenotype. Points indicate prevalence ratios per 1‐SD increment, and horizontal bars indicate 95% confidence intervals. Results are shown separately for CHNS and NHANES. All models were adjusted for age, urban/rural residence, education, alcohol drinking and total energy intake (CHNS) or age, race/ethnicity, education, smoking and total energy intake (NHANES), using the same, common, BMI‐free covariate specification for all five indices so that estimates are directly comparable. BMI‐adjusted estimates for HOMA‐IR, TyG and TG/HDL‐C are shown separately as secondary sequential‐model results in Supporting Figure S1 and are not compared against TyG‐BMI or METS‐IR, because BMI is embedded in the latter two index formulas. Fully adjusted regression models included 1721 CHNS participants and 2799 NHANES participants with complete covariate data. CHNS, China Health and Nutrition Survey; NHANES, National Health and Nutrition Examination Survey; PR, prevalence ratio; SD, standard deviation; CI, confidence interval; BMI, body mass index.

Quartile‐based analyses showed positive gradients for all five indices. In CHNS, Q4‐versus‐Q1 PRs ranged from 1.234 (95% CI, 1.165–1.308) for HOMA‐IR to 1.537 (95% CI, 1.423–1.659) for METS‐IR. In NHANES, the corresponding PRs ranged from 1.564 (95% CI, 1.451–1.686) for TyG‐BMI to 1.648 (95% CI, 1.530–1.776) for METS‐IR. *p* for trend was < 0.001 for every index in both datasets. Detailed quartile‐specific estimates are provided in Supporting Table S3, and the associations remained positive across the sequential adjustment models shown in Supporting Figure S1.

### 3.3. Exploratory Internal Discrimination

For the primary component‐defined MUP outcome, exploratory internal discrimination varied between the two datasets. In CHNS, HOMA‐IR had an AUC of 0.690 (95% CI, 0.658–0.722). TyG, TG/HDL‐C, TyG‐BMI and METS‐IR had higher AUCs than HOMA‐IR, with METS‐IR showing the highest AUC of 0.823 (95% CI, 0.799–0.847). All four pairwise comparisons with HOMA‐IR had *p* < 0.001 (Table 3; Figure 3).

**TABLE 3:** Exploratory internal discrimination for component‐defined metabolically unhealthy phenotype.

Index	CHNS		NHANES	
	AUC (95% CI)	DeLong *P* vs. HOMA‐IR	AUC (95% CI)	DeLong *P* vs. HOMA‐IR
HOMA‐IR	0.690 (0.658, 0.722)	Reference	0.796 (0.776, 0.816)	Reference
TyG	0.814 (0.791, 0.837)	< 0.001	0.792 (0.773, 0.810)	0.703
TG/HDL‐C	0.806 (0.782, 0.829)	< 0.001	0.766 (0.746, 0.787)	0.017
TyG‐BMI	0.810 (0.784, 0.836)	< 0.001	0.789 (0.767, 0.811)	0.560
METS‐IR	0.823 (0.799, 0.847)	< 0.001	0.794 (0.773, 0.815)	0.867

*Note:* AUC estimates are shown for the primary component‐defined metabolically unhealthy phenotype. ROC analyses were empirical internal discrimination analyses. NHANES ROC estimates did not incorporate survey weights, strata or primary sampling units. HOMA‐IR was used as the reference index for DeLong comparisons. A survey‐design‐aware companion analysis incorporating WTSAF8YR, SDMVSTRA and SDMVPSU is reported separately in Supporting Table S11 and Supporting Figure S7. Because several index formulas mathematically share glucose, triglyceride, HDL‐C or adiposity‐related information with the metabolic components defining MUP, these AUC differences represent concurrent classification under substantial predictor–outcome component overlap rather than evidence that one index has superior or independent metabolic‐risk identification ability. AUC, area under the receiver operating characteristic curve; HOMA‐IR, homeostasis model assessment of insulin resistance.

Abbreviations: CHNS, China Health and Nutrition Survey; CI, confidence interval; NHANES, National Health and Nutrition Examination Survey; ROC, receiver operating characteristic.

**FIGURE 3:**
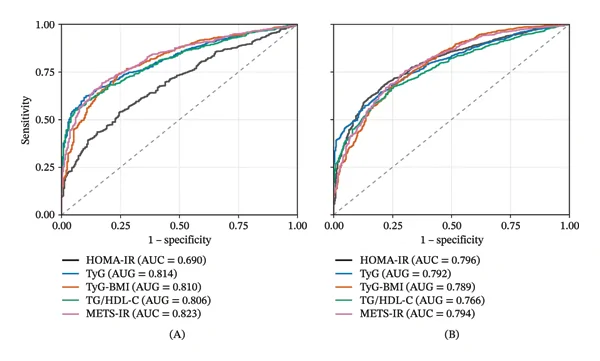
Exploratory receiver operating characteristic curves for component‐defined metabolically unhealthy phenotype. (A and B) Empirical receiver operating characteristic curves for component‐defined metabolically unhealthy phenotype in CHNS and NHANES respectively. Curves describe exploratory internal discrimination and should not be interpreted as validation of clinical diagnostic or prediction thresholds. The CHNS ROC analysis included 1751 participants, and the NHANES ROC analysis included 2953 participants. CHNS, China Health and Nutrition Survey; NHANES, National Health and Nutrition Examination Survey; ROC, receiver operating characteristic; AUC, area under the receiver operating characteristic curve.

In NHANES, HOMA‐IR had an AUC of 0.796 (95% CI, 0.776–0.816). The AUCs of TyG, TyG‐BMI and METS‐IR were 0.792, 0.789 and 0.794, respectively, and did not differ significantly from that of HOMA‐IR (DeLong *p* = 0.703, *p* = 0.560 and *p* = 0.867 respectively). TG/HDL‐C had a lower AUC of 0.766 (95% CI, 0.746–0.787) than HOMA‐IR (DeLong *p* = 0.017). Detailed empirical ROC results for the primary phenotype and blood pressure abnormality, including internally derived Youden cut‐offs, sensitivity and specificity, are reported in Supporting Table S11. Results for the alternative phenotype definitions are shown in Supporting Figures S2–S3. All ROC‐derived cut‐offs were treated as exploratory internal thresholds and not as validated clinical cut‐offs.

A survey‐design‐aware companion analysis in NHANES, incorporating WTSAF8YR, SDMVSTRA and SDMVPSU, produced broadly similar weighted AUCs: HOMA‐IR 0.789 (95% CI, 0.766–0.812), TyG 0.794 (0.770–0.815), TG/HDL‐C 0.777 (0.754–0.797), TyG‐BMI 0.774 (0.747–0.800) and METS‐IR 0.780 (0.751–0.805) (Supporting Table S11; Supporting Figure S7). Because a survey‐weighted paired DeLong test was not implemented, these weighted AUCs are not accompanied by formal pairwise comparison *P* values and are interpreted as design‐based descriptive estimates rather than population‐level diagnostic validation.

### 3.4. Composite Metabolic Dysregulation Burden and Consistency Across CHNS and NHANES

The composite metabolic dysregulation burden score provided a complementary continuous representation of multidomain metabolic variation. It was constructed in the final analytic samples of 1751 CHNS participants and 2953 NHANES participants using adverse‐direction standardised measures of adiposity, blood pressure, glycaemic status, TG and HDL‐C.

All five indices were positively correlated with the composite metabolic dysregulation burden score in both datasets. METS‐IR had the highest Spearman correlation in CHNS and NHANES (rho = 0.759 and rho = 0.789 respectively). For the covariate‐adjusted beta coefficient per 1‐SD increment, computed from the same common, BMI‐free specification used for the main association analyses, METS‐IR was largest in CHNS (0.415), whereas TyG was largest in NHANES (0.424), with METS‐IR second (0.405) in NHANES. TyG‐BMI ranked second for Spearman correlation in both datasets and second for adjusted beta in CHNS (0.385), but third for adjusted beta in NHANES (0.380). HOMA‐IR had the lowest Spearman correlations and adjusted beta coefficients in both datasets, while TG/HDL‐C showed intermediate patterns throughout. All adjusted associations had *p* < 0.001 (Supporting Table S6).

A component‐exclusion analysis showed that removing each index’s formula‐overlapping domains substantially reduced its correlation with the burden score, most markedly for METS‐IR (Spearman’s rho declining from 0.759 to 0.219 in CHNS and from 0.789 to 0.127 in NHANES) and TyG‐BMI (from 0.722 to 0.585 in CHNS and from 0.747 to 0.455 in NHANES), with smaller reductions for TyG, TG/HDL‐C and HOMA‐IR (Supporting Table S19). These findings indicate that much of the full‐score alignment for the BMI‐ and glucose/triglyceride‐containing indices reflects formula‐to‐domain overlap rather than independent concordance with the composite burden score.

Rank consistency across CHNS and NHANES was classified as high for METS‐IR, TyG‐BMI and TG/HDL‐C. TyG was classified as having moderate rank consistency: although its Spearman correlation and exploratory AUC ranks were similar between datasets, its adjusted‐beta rank differed by two positions, moving from third in CHNS to first in NHANES. HOMA‐IR was classified as having low rank consistency because its composite burden alignment rank was similar between the two datasets, whereas its exploratory AUC rank differed substantially. These rankings summarised relative within‐dataset patterns across composite burden alignment, adjusted associations and exploratory discrimination and were interpreted descriptively within the two datasets (Figure 4; Supporting Tables S6–S7).

**FIGURE 4:**
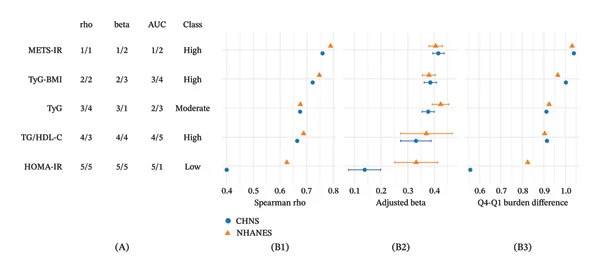
Alignment with composite metabolic dysregulation burden and consistency across CHNS and NHANES. (A) Rank consistency between CHNS and NHANES across empirical index–burden alignment, adjusted beta for composite burden and exploratory AUC for component‐defined metabolically unhealthy phenotype. Ranks are shown as CHNS/NHANES, and rank 1 indicates the strongest positive signal within each database. (B1) Spearman correlations between each surrogate index and the composite metabolic dysregulation burden score. (B2) Covariate‐adjusted beta coefficients for the composite burden score per 1‐SD higher index, with horizontal bars indicating 95% confidence intervals. (B3) The difference in mean composite burden score between the highest and lowest index quartiles. The composite burden score was constructed as the mean of adverse‐direction standardised metabolic domains, including adiposity, blood pressure, glycaemic status, triglycerides and HDL‐C. CHNS composite burden analyses included 1751 participants, and NHANES composite burden analyses included 2953 participants. CHNS, China Health and Nutrition Survey; NHANES, National Health and Nutrition Examination Survey; HDL‐C, high‐density lipoprotein cholesterol; SD, standard deviation; CI, confidence interval.

### 3.5. Correlations With HOMA‐IR

All four noninsulin surrogate indices were positively correlated with HOMA‐IR in both datasets. In the CHNS final analytic sample, Spearman correlation coefficients ranged from 0.300 for TG/HDL‐C to 0.427 for TyG. In NHANES, the coefficients ranged from 0.449 for TG/HDL‐C to 0.592 for METS‐IR. These correlations are summarised together with the association and exploratory discrimination estimates in Figure 5.

**FIGURE 5:**
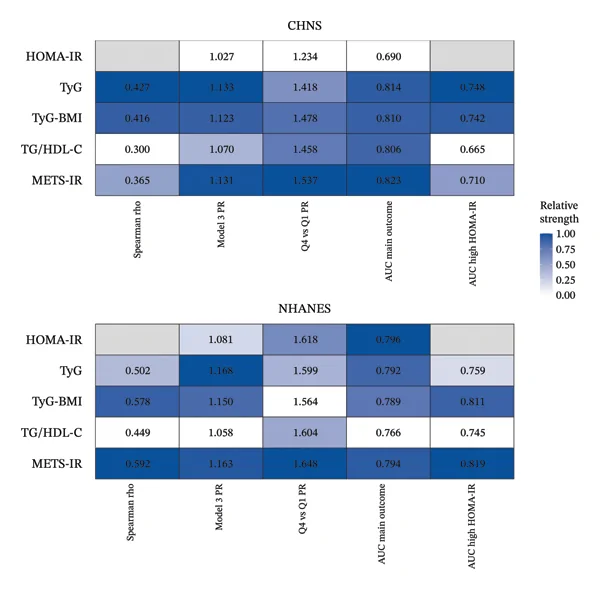
Relative strength summary across association and discrimination evidence. Values shown in cells are raw estimates. Colours indicate column‐wise relative strength within each database, and grey cells indicate not applicable estimates. Spearman rho represents the empirical correlation of each noninsulin surrogate index with HOMA‐IR in the final analytic samples of CHNS and NHANES. Model 3 PR indicates the common, fully adjusted, BMI‐free prevalence ratios for the component‐defined metabolically unhealthy phenotype, applied identically to all five indices. Q4 vs. Q1 PR compares the highest versus lowest index quartile under the same common model. AUC for the main outcome indicates exploratory internal discrimination for the component‐defined metabolically unhealthy phenotype. AUC for high HOMA‐IR indicates exploratory discrimination for high HOMA‐IR; HOMA‐IR itself was not evaluated for this outcome. CHNS, China Health and Nutrition Survey; NHANES, National Health and Nutrition Examination Survey; PR, prevalence ratio; AUC, area under the receiver operating characteristic curve; Q, quartile.

To evaluate whether the burden‐score correlations were driven by formula‐to‐domain overlap, a component‐exclusion analysis removed, for each index, the burden‐score domains entering its own formula (e.g. the glycaemic domain for HOMA‐IR, or the glycaemic, triglyceride and adiposity domains for METS‐IR) and recomputed the Spearman correlation between the index and the domain‐excluded residual burden score.

### 3.6. Sensitivity and Supportive Subgroup Analyses

Sensitivity analyses using alternative phenotype definitions supported the main findings. Associations remained positive for all five indices when MUP was defined using at least three abnormal metabolic components or a common 85‐cm waist‐circumference cut‐off in both databases (Supporting Table S10). A genuinely harmonised phenotype, applying an identical 85‐cm cut‐off and excluding medication‐based components in both databases, likewise showed positive associations for all five indices (CHNS *n* = 1,721, 1364 events; NHANES *n* = 2,799, 2390 events), as did a uniform BMI ≥ 25 kg/m^2^ inclusion sensitivity (Supporting Table S15).

Component‐specific results varied according to formula‐to‐outcome overlap. Blood pressure abnormality, the only component absent from all five index formulas, was positively associated with every index in both datasets, including HOMA‐IR (CHNS PR = 1.032, 95% CI 1.009–1.055, *p* = 0.006; NHANES PR = 1.092, 95% CI 1.054–1.131, *p* < 0.001) (Supporting Tables S4–S5). Associations with MUP remained positive for TyG, TG/HDL‐C, TyG‐BMI and METS‐IR within the obesity strata of both datasets; the HOMA‐IR association was not statistically significant in the CHNS obesity stratum but remained positive in NHANES (Supporting Table S9; distributions across increasing metabolic‐abnormality counts are shown in Supporting Figure S5). A strict nonoverlap definition, which removed each index’s formula‐overlapping components and required at least two of the remaining components to be abnormal, again produced positive associations for HOMA‐IR, TyG, TG/HDL‐C and TyG‐BMI in both datasets; a residual outcome could not be defined for METS‐IR because only blood pressure remained once its overlapping components were removed (Supporting Table S13).

The findings were also robust to how the indices were modelled. Log‐transformation, interquartile‐range standardisation and winsorisation all produced associations consistent in direction with the primary per‐1‐SD estimates (Supporting Table S14), while restricted cubic spline models on the raw index scale detected statistically significant departures from linearity for every index in both datasets (all nonlinear Wald *p* < 0.001); PRs rose steeply at lower index values before plateauing at higher values, a pattern that motivates reporting the complementary quartile‐based analyses alongside the per‐1‐SD estimates (Supporting Table S21; Supporting Figure S8). Excluding participants with diabetes (CHNS *n* = 1473; NHANES *n* = 2153) or modelling the number of abnormal components as a count outcome did not materially change the direction or significance of any association (Supporting Tables S14–S17). Finally, E‐value analyses indicated that an unmeasured confounder would need a stronger association with both exposure and outcome than observed to fully explain the associations away, with point estimates from 1.19 to 2.68 and confidence‐limit estimates from 1.11 to 2.43 across indices, databases and contrasts (Supporting Table S18).

Supportive age‐group and BMI‐category analyses (age groups and BMI categories as defined in Methods) showed positive associations in most strata, although association magnitudes varied and interaction tests indicated heterogeneity for several indices; full results are presented in Supporting Tables S8–S9 and Supporting Figure S4.

## 4. Discussion

In this cross‐sectional analysis of adult women with overweight or obesity, all five surrogate indices were positively associated with component‐defined MUP in both CHNS and NHANES. Positive associations were observed in the per 1‐SD and quartile‐based models and under both alternative phenotype definitions. All five indices were also positively associated with blood pressure abnormality, extending the observed pattern beyond glucose and lipid components. The composite burden score further linked these indices to multidomain metabolic variation beyond the binary MUP outcome. Together, these findings place insulin resistance–related measures within a broader pattern of endocrine‐metabolic dysfunction among women with excess adiposity. Their biological relevance can be considered through the coordinated actions of insulin on adipose tissue, hepatic glucose and lipid metabolism and vascular function.

The five indices incorporate different metabolic measures and capture distinct dimensions of insulin resistance. HOMA‐IR reflects fasting insulin–glucose homeostasis and requires insulin measurement [5]. TyG represents triglyceride–glucose coupling and has been evaluated against more direct measures of insulin sensitivity and in studies of metabolic syndrome and related phenotypes [6, 7, 20, 21]. TG/HDL‐C represents a lipid ratio whose association with insulin resistance may vary across racial, ethnic and sex‐specific populations [22–25]. TyG‐BMI combines TyG with general adiposity [10, 26, 27], whereas METS‐IR integrates glycaemic, lipid and adiposity‐related information [11, 28].

The metabolic dysregulation burden analysis provided a complementary continuous perspective on multidomain metabolic variation. METS‐IR and TyG‐BMI showed the strongest empirical Spearman correlation with the composite burden score in both datasets, whereas for the covariate‐adjusted association, TyG rather than METS‐IR or TyG‐BMI showed the largest coefficient in NHANES. This pattern is biologically consistent with the shared formula composition of the burden score and the two adiposity‐integrated indices [1]. Visceral and ectopic fat accumulation increases free fatty acid flux to the liver, a pathway linked to hepatic and peripheral insulin resistance and considered in more detail below [29, 30]. Because TyG‐BMI and METS‐IR incorporate BMI alongside glucose and TG, they mechanistically track this same upstream adipose tissue dysfunction that drives the glycaemic, lipid and adiposity domains of the burden score. HOMA‐IR, in contrast, is confined to the fasting insulin–glucose axis and does not capture this broader lipid‐adiposity pathway, which explains its comparatively weaker alignment with the multidomain score even though it remains an established measure of insulin resistance in its own right.

These differences can be considered in the broader context of variation in metabolic responses to excess adiposity. In a controlled weight‐gain study of participants with obesity matched for BMI and fat mass, similar weight gain was accompanied by deterioration in hepatic, skeletal muscle and adipose tissue insulin sensitivity in metabolically abnormal participants, but not in metabolically normal participants; differences in adipose tissue lipogenic responses suggested a possible role for lipid‐storage adaptation [31]. This provides a biological rationale for considering metabolic function alongside the amount of adiposity. In the present study, the associations of indices containing different combinations of insulin, glucose, lipids and BMI are consistent with multiple aspects of metabolic dysregulation accompanying MUP. However, these indices do not directly measure tissue‐specific insulin sensitivity or adipose tissue function. Moreover, several constituent variables also enter the MUP definition [4, 32].

The adipose tissue‐liver pathway introduced above can be traced in more detail through the interaction between adipose tissue insulin action and hepatic substrate metabolism. Rodent experiments have shown that insulin suppresses hepatic glucose production partly by inhibiting adipose tissue lipolysis, thereby reducing hepatic acetyl‐CoA availability and pyruvate carboxylase activity; disruption of this pathway links adipose tissue inflammation to increased hepatic glucose production [33]. Human metabolic studies extend this pathway to lipid regulation. Among participants with obesity matched for visceral fat, greater intrahepatic fat was associated with lower hepatic, skeletal muscle and adipose tissue insulin sensitivity and increased hepatic VLDL‐triglyceride secretion [34], consistent with the coexistence of impaired insulin action, dysglycaemia and hypertriglyceridaemia captured by the insulin‐, glucose‐ and triglyceride‐based measures used in our study. Lipoprotein metabolism further connects the lipid components. A tracer study in healthy men demonstrated that triglyceride enrichment of HDL accelerated the metabolic clearance of HDL apoA‐I [35], a mechanism that helps explain why elevated TG and reduced HDL‐C may occur together and that provides context for the associations of TG/HDL‐C and METS‐IR with MUP.

Insulin also participates in vascular regulation, which connects our blood pressure findings to the same biological framework. In spontaneously hypertensive rats, insulin resistance was accompanied by impaired PI3K‐dependent nitric oxide production and enhanced MAPK‐dependent endothelin‐1 secretion, linking altered insulin signalling to an imbalance between endothelial vasodilator and vasoconstrictor activity [36]. The positive associations between all five indices and blood pressure abnormality can therefore be considered alongside the glucose and lipid findings within a broader framework of metabolic and vascular dysfunction. Together, these mechanisms provide a biological framework for understanding why women within the same broad weight category may exhibit different combinations and degrees of metabolic abnormality.

Beyond these mechanistic links, because TyG, TG/HDL‐C, TyG‐BMI and METS‐IR are derived from routinely measured biochemical and anthropometric variables, the findings are also relevant to population‐level characterisation of metabolic heterogeneity among women with overweight or obesity. Previous population studies have evaluated TyG and related indices in relation to metabolic syndrome, diabetes and other insulin‐resistance‐related phenotypes [20, 21, 26–28], while recent evidence has highlighted heterogeneity between metabolically healthy and unhealthy obesity phenotypes in adult populations [37, 38]. The consistency of the observed associations across CHNS and NHANES similarly reinforces their relevance across two distinct population datasets, although the positive direction of the associations does not establish generalisability to other populations, and the two databases were less concordant in their exploratory ROC‐based discrimination results; NHANES provided an independent replication of the association pattern rather than external validation of a clinical diagnostic or prediction model.

Evidence from women further supports the endocrine‐metabolic relevance of insulin resistance. In the Women’s Health Initiative Observational Study, a prospective nested case‐control analysis found that higher HOMA‐IR was associated with incident diabetes in postmenopausal women [39]. HOMA‐IR offers an epidemiological measure of fasting insulin–glucose homeostasis, while the euglycaemic–hyperinsulinaemic clamp provides a more direct physiological assessment of insulin sensitivity but is less feasible in large population studies [40, 41]. In our study, the associations of both insulin‐dependent and noninsulin indices with MUP across CHNS and NHANES highlight variation in metabolic health among women who already meet criteria for overweight or obesity. Insulin‐related, glycaemic and lipid information, alongside adiposity, can therefore help describe differences in the clustering of metabolic abnormalities within this population. This focus on metabolic function is relevant to understanding why a shared weight classification can encompass substantially different endocrine‐metabolic profiles.

### 4.1. Strengths and Limitations

This study has several strengths. First, it focused specifically on adult women with overweight or obesity, a population in whom metabolic heterogeneity is relevant to epidemiological research and population health. Second, CHNS and NHANES were analysed separately as discovery and independent replication datasets, preserving their distinct sampling and analytic structures; the complex survey design was incorporated into the NHANES regression analyses. Third, the study compared five indices representing different combinations of insulin, glucose, lipid and adiposity‐related information, including HOMA‐IR as an insulin‐dependent reference surrogate. Fourth, the analyses included per 1‐SD associations, quartile‐based gradients, alternative phenotype definitions, component‐specific outcomes, supportive age and BMI subgroup analyses and exploratory internal discrimination. The multidomain metabolic burden score provided a complementary continuous representation of metabolic variation, while the component‐specific analyses allowed formula‐to‐outcome overlap to be examined explicitly.

Several limitations should be considered. First, the cross‐sectional design precludes assessment of temporality and causal direction. Second, several index formulas share glucose, triglyceride, HDL‐C or adiposity‐related information with components of the MUP definition. This formula‐to‐outcome overlap may strengthen the observed associations and internal discrimination. Although the Discussion situates this overlap within a plausible shared biological pathway, the constructed multidomain metabolic burden score incorporated some of the same domains and therefore did not provide statistically independent validation of that pathway. Third, HOMA‐IR is itself a surrogate index, and neither dataset included a euglycaemic–hyperinsulinaemic clamp measure; the study therefore cannot determine which index most accurately reflects biological insulin resistance. Fourth, the ROC analyses were internally derived and empirical, and the NHANES ROC estimates did not incorporate the complex survey design. The resulting AUCs and Youden cut‐offs should not be interpreted as nationally representative diagnostic performance estimates or clinical thresholds. Fifth, the BMI‐stratified subgroup estimates for TyG‐BMI and METS‐IR, including the markedly larger PRs observed in normal‐weight strata (Supporting Table S9; Supporting Figure S4), should be interpreted as exploratory scale or construct sensitivity analyses rather than conventional effect modification, because BMI defines the strata while also being a multiplicative or additive component of both index formulas. Finally, differences in source population, operational definitions, sampling design and laboratory procedures limit direct numerical comparison between CHNS and NHANES and the generalisability of the findings to other populations.

## 5. Conclusions

Among adult women with overweight or obesity, higher HOMA‐IR, TyG, TG/HDL‐C, TyG‐BMI and METS‐IR values were associated with component‐defined MUP in both CHNS and NHANES. Their associations with composite metabolic burden and blood pressure abnormality further situated these measures within a broader pattern of metabolic dysfunction. Viewed alongside established biological links between insulin action, adipose tissue function, hepatic glucose and lipid metabolism and vascular regulation, these findings underscore the relevance of insulin resistance–related disturbances to metabolic heterogeneity in women with excess adiposity. Characterising this heterogeneity requires attention to metabolic function and the clustering of abnormalities alongside body size.

**Nomenclature**
AUC: Area under the receiver operating characteristic curve
BMI: Body mass index
CHNS: China Health and Nutrition Survey
CI: Confidence interval
HbA1c: Glycated haemoglobin
HDL‐C: High‐density lipoprotein cholesterol
HOMA‐IR: Homeostasis model assessment of insulin resistance
IQR: Interquartile range
METS‐IR: Metabolic score for insulin resistance
MUP: Metabolically unhealthy phenotype
NHANES: National Health and Nutrition Examination Survey
PR: Prevalence ratio
PSU: Primary sampling unit
ROC: Receiver operating characteristic
SD: Standard deviation
TG: Triglycerides
TG/HDL‐C: Triglyceride‐to‐high‐density lipoprotein cholesterol ratio
TyG: Triglyceride–glucose index
TyG‐BMI: Triglyceride–glucose body mass index
WC: Waist circumference

## Author Contributions

Xiaoye Zhu conceived and designed the study, performed data curation and statistical analysis and drafted the manuscript. Jinghui Zou, Hong Zhu and Honghong Wang contributed to the interpretation of the results. Yanfei Jin and Yisheng Zhang critically revised the manuscript for important intellectual content.

## Funding

This work was supported by the Key Research and Development Project of Ningbo (No. 2023Z185) and the Huili Medical and Health Technology Project (No. 2025MS030).

## Disclosure

All authors read and approved the final manuscript. The funders had no role in the design of the study; the collection, analysis, or interpretation of data; the writing of the manuscript; or the decision to submit the article for publication.

## Ethics Statement

The China Health and Nutrition Survey was approved by the institutional review committees of the National Institute of Nutrition and Food Safety, the Chinese Center for Disease Control and Prevention, the University of North Carolina at Chapel Hill, and the China‐Japan Friendship Hospital, Ministry of Health. The National Health and Nutrition Examination Survey was approved by the National Center for Health Statistics Research Ethics Review Board. Written informed consent was obtained from all participants in the original surveys. The present study was a secondary analysis of de‐identified CHNS and NHANES data and was conducted in accordance with the Declaration of Helsinki and relevant database data‐use policies. No additional ethics approval was required for the present analysis.

## Consent

Please see the Ethics Statement.

## Conflicts of Interest

The authors declare no conflicts of interest.

## Supporting Information

Additional supporting information can be found online in the Supporting Information section.

## Supporting information


**Supporting Information 1** Supporting Table S1. Operational definitions of metabolic abnormality components in CHNS and NHANES. Supporting Table S2. Database‐specific standard deviations and distributions of surrogate indices. Supporting Table S3. Quartile associations with the component‐defined metabolically unhealthy phenotype. Supporting Table S4. Associations with blood pressure abnormality as a conservative component outcome. Supporting Table S5. Component‐specific associations of surrogate indices with metabolic abnormality components. Supporting Table S6. Alignment between surrogate indices and the composite metabolic dysregulation burden score. Supporting Table S7. Cross‐database rank‐consistency summary. Supporting Table S8. Age‐stage stratified associations and interaction tests. Supporting Table S9. Full BMI‐spectrum sensitivity analyses. Supporting Table S10. Sensitivity analyses using alternative phenotype definitions. Supporting Table S11. Exploratory ROC/AUC details for the primary phenotype and blood pressure abnormality. Supporting Table S12. Sensitivity analysis excluding known pregnancy in CHNS. Supporting Table S13. Strict nonoverlap associations after excluding formula‐overlapping components. Supporting Table S14. Robustness of associations to the functional form of the surrogate indices. Supporting Table S15. Associations with a harmonised metabolically unhealthy phenotype (uniform waist cut‐off, medication excluded). Supporting Table S16. Associations after excluding participants with diabetes. Supporting Table S17. Associations with the number of metabolic abnormalities modelled as a count outcome. Supporting Table S18. E‐values for the associations of surrogate indices with the metabolically unhealthy phenotype. Supporting Table S19. Alignment of surrogate indices with the composite burden score before and after removing formula‐overlapping domains. Supporting Table S20. Comparison of model‐based (naïve, i.i.d.) and community‐clustered robust standard errors for the CHNS analyses (primary, BMI‐excluded models). Supporting Table S21. Nonlinearity of index–outcome associations: restricted cubic spline Wald tests. Supporting Figure S1. Associations across four sequential adjustment models. Supporting Figure S2. ROC analyses for alternative phenotype definitions in CHNS. Supporting. Figure S3. ROC analyses for alternative phenotype definitions in NHANES. Supporting Figure S4. Age‐stage and BMI‐category subgroup analyses. Supporting Figure S5. Distribution of surrogate indices according to metabolic abnormality burden. Supporting Figure S6. Standardised differences in baseline characteristics by metabolic phenotype. Supporting Figure S7. Survey‐design‐aware NHANES receiver operating characteristic curves for component‐defined metabolically unhealthy phenotype. Supporting Figure S8. Restricted cubic spline associations between surrogate indices and component‐defined metabolically unhealthy phenotype.

**Supporting Information 2** Revised STROBE.

## Data Availability

The datasets analysed during the current study are available from the China Health and Nutrition Survey repository (https://chns.cpc.unc.edu/data/datasets/) and the National Health and Nutrition Examination Survey repository (https://wwwn.cdc.gov/nchs/nhanes/). Access to CHNS data is subject to the applicable data‐use requirements of the CHNS repository. NHANES data are publicly available.
