# Supplementary material for: Associations of Insulin Resistance Surrogate Indices With Component‐Defined Metabolically Unhealthy Phenotype Among Women With Overweight or Obesity: A Cross‐Sectional Analysis of CHNS and NHANES
Source: Int J Endocrinol. 2026 Oct 14;2026:5660424. doi: 10.1155/ije/5660424 (PMC13662632; doi:10.1155/ije/5660424)
Supplement: Supplementary file 1 [file IJE-2026-5660424-s002.pdf]

Supplementary Table S1. Operational definitions of metabolic abnormality components in CHNS and NHANES

Component	CHNS operational definition	NHANES operational definition
Blood pressure abnormality	Systolic blood pressure ≥130 mmHg, diastolic blood pressure ≥85 mmHg, or available hypertension history. Medication information was not consistently available in the CHNS analytic dataset.	Systolic blood pressure ≥130 mmHg, diastolic blood pressure ≥85 mmHg, self-reported hypertension, or antihypertensive medication use.
Dysglycaemia	Fasting glucose ≥5.6 mmol/L, HbA1c ≥5.7%, or available diabetes-related questionnaire information. Diabetes medication or history information was not consistently available in the CHNS analytic dataset.	Fasting glucose ≥5.6 mmol/L, HbA1c ≥5.7%, self-reported diabetes, insulin use, or antidiabetic medication use.
Elevated triglycerides	Triglycerides ≥1.7 mmol/L.	Triglycerides ≥1.7 mmol/L.
Reduced HDL-C	HDL-C <1.29 mmol/L.	HDL-C <1.29 mmol/L.
Elevated waist circumference	Waist circumference ≥80 cm.	Waist circumference ≥88 cm.
Component-defined metabolically unhealthy phenotype	At least two abnormal metabolic components.	At least two abnormal metabolic components.

Note. Operational definitions were based on the variables available in each database and the harmonized analytic definitions used in this study. Glucose, triglycerides and HDL-C were harmonized before analysis when required.
Abbreviations: CHNS, China Health and Nutrition Survey; HbA1c, glycated haemoglobin; HDL-C, high-density lipoprotein cholesterol; NHANES, National Health and Nutrition Examination Survey.

Supplementary Table S2. Database-specific standard deviations and distributions of surrogate indices

Index	CHNS SD used for scaling	CHNS distribution	NHANES SD used for scaling	NHANES distribution
HOMA-IR	6.849	2.95 [1.96, 4.53]	7.165	3.18 [2.01, 5.19]
TyG	0.693	8.82 (0.69)	0.668	8.60 (0.67)
TG/HDL-C	1.706	1.12 [0.71, 1.85]	1.312	0.81 [0.51, 1.29]
TyG-BMI	31.088	231.73 [215.44, 254.33]	64.111	271.53 [237.64, 319.27]
METS-IR	5.922	39.03 [35.92, 43.27]	11.869	46.11 [39.84, 55.15]

Note. Per 1-SD estimates used database-specific standard deviations. Distributions are unweighted descriptive summaries of the frozen eligible populations (CHNS n=1,751; NHANES n=2,953) and are shown as mean (SD) or median [Q1, Q3].

Abbreviations: CHNS, China Health and Nutrition Survey; NHANES, National Health and Nutrition Examination Survey; SD, standard deviation.

Supplementary Table S3. Quartile associations with the component-defined metabolically unhealthy phenotype

Index	Quartile	CHNS				NHANES			
		Range	PR (95% CI)	P value	P for trend	Range	PR (95% CI)	P value	P for trend
HOMA-IR	Q1	[0.02, 1.96]	Reference	Reference	—	[0.03, 2.01]	Reference	Reference	—
	Q2	(1.96, 2.95]	1.077 (1.003, 1.157)	0.041	—	(2.01, 3.18]	1.304 (1.184, 1.436)	<0.001	—
	Q3	(2.95, 4.53]	1.170 (1.098, 1.245)	<0.001	—	(3.18, 5.19]	1.553 (1.416, 1.703)	<0.001	—
	Q4	(4.53, 125.07]	1.234 (1.165, 1.308)	<0.001	<0.001	(5.19, 140.54]	1.618 (1.486, 1.762)	<0.001	<0.001
TyG	Q1	[7.12, 8.34]	Reference	Reference	—	[6.19, 8.13]	Reference	Reference	—
	Q2	(8.34, 8.77]	1.158 (1.072, 1.252)	<0.001	—	(8.13, 8.57]	1.275 (1.165, 1.396)	<0.001	—
	Q3	(8.77, 9.25]	1.383 (1.289, 1.485)	<0.001	—	(8.57, 9.01]	1.479 (1.357, 1.613)	<0.001	—
	Q4	(9.25, 11.63]	1.418 (1.322, 1.521)	<0.001	<0.001	(9.01, 12.84]	1.599 (1.481, 1.727)	<0.001	<0.001
TyG-BMI	Q1	[172.4, 215.4]	Reference	Reference	—	[164.2, 237.6]	Reference	Reference	—
	Q2	(215.4, 231.7]	1.305 (1.209, 1.408)	<0.001	—	(237.6, 271.5]	1.285 (1.175, 1.405)	<0.001	—
	Q3	(231.7, 254.3]	1.448 (1.347, 1.556)	<0.001	—	(271.5, 319.3]	1.415 (1.300, 1.541)	<0.001	—
	Q4	(254.3, 392.4]	1.478 (1.373, 1.590)	<0.001	<0.001	(319.3, 631.5]	1.564 (1.451, 1.686)	<0.001	<0.001
TG/HDL-C	Q1	[0.12, 0.71]	Reference	Reference	—	[0.06, 0.51]	Reference	Reference	—
	Q2	(0.71, 1.12]	1.195 (1.106, 1.290)	<0.001	—	(0.51, 0.81]	1.269 (1.156, 1.393)	<0.001	—
	Q3	(1.12, 1.85]	1.391 (1.293, 1.497)	<0.001	—	(0.81, 1.29]	1.447 (1.331, 1.573)	<0.001	—
	Q4	(1.85, 27.42]	1.458 (1.359, 1.566)	<0.001	<0.001	(1.29, 45.07]	1.604 (1.471, 1.749)	<0.001	<0.001
METS-IR	Q1	[26.7, 35.9]	Reference	Reference	—	[27.7, 39.8]	Reference	Reference	—
	Q2	(35.9, 39.0]	1.320 (1.221, 1.428)	<0.001	—	(39.8, 46.1]	1.311 (1.202, 1.431)	<0.001	—
	Q3	(39.0, 43.3]	1.487 (1.378, 1.605)	<0.001	—	(46.1, 55.1]	1.453 (1.335, 1.581)	<0.001	—
	Q4	(43.3, 70.2]	1.537 (1.423, 1.659)	<0.001	<0.001	(55.1, 116.4]	1.648 (1.530, 1.776)	<0.001	<0.001

Note. Q1 was the reference group for each index. Estimates are prevalence ratios under the common BMI-free specification, with community-clustered robust variance in CHNS and complex-survey estimation in NHANES. Regression complete cases were CHNS n=1,721 (1,473 events) and NHANES n=2,799 (2,348 events). P for trend was obtained by modelling quartile as an ordinal term.

Abbreviations: CHNS, China Health and Nutrition Survey; CI, confidence interval; NHANES, National Health and Nutrition Examination Survey; PR, prevalence ratio; Q, quartile.

Supplementary Table S4. Associations with blood pressure abnormality as a conservative component outcome

Index	CHNS				NHANES			
Index	PR (95% CI)	P value	n	BP events	PR (95% CI)	P value	n	BP events
HOMA-IR	1.032 (1.009, 1.055)	0.006	1721	932	1.092 (1.054, 1.131)	<0.001	2799	1551
TyG	1.124 (1.083, 1.167)	<0.001	1721	932	1.152 (1.105, 1.201)	<0.001	2799	1551
TyG-BMI	1.177 (1.135, 1.219)	<0.001	1721	932	1.218 (1.173, 1.265)	<0.001	2799	1551
TG/HDL-C	1.078 (1.054, 1.102)	<0.001	1721	932	1.064 (1.020, 1.109)	0.006	2799	1551
METS-IR	1.163 (1.124, 1.204)	<0.001	1721	932	1.221 (1.177, 1.267)	<0.001	2799	1551

Note. Blood pressure abnormality was evaluated as a conservative component outcome because blood pressure does not directly enter any surrogate-index formula. Estimates are prevalence ratios per 1-SD increment under the common BMI-free specification. CHNS used community-clustered robust variance (n=1,721; 932 events); NHANES used the fasting subsample survey design (n=2,799; 1,551 events).

Abbreviations: BP, blood pressure; CHNS, China Health and Nutrition Survey; CI, confidence interval; NHANES, National Health and Nutrition Examination Survey; PR, prevalence ratio; SD, standard deviation.

Supplementary Table S5. Component-specific associations of surrogate indices with metabolic abnormality components

Component outcome	Index	Overlap category	CHNS		NHANES	
			PR (95% CI)	P value	PR (95% CI)	P value
BP abnormality	HOMA-IR	None	1.032 (1.009, 1.055)	0.006	1.092 (1.054, 1.131)	<0.001
	TyG	None	1.124 (1.083, 1.167)	<0.001	1.152 (1.105, 1.201)	<0.001
	TyG-BMI	None	1.177 (1.135, 1.219)	<0.001	1.218 (1.173, 1.265)	<0.001
	TG/HDL-C	None	1.078 (1.054, 1.102)	<0.001	1.064 (1.020, 1.109)	0.006
	METS-IR	None	1.163 (1.124, 1.204)	<0.001	1.221 (1.177, 1.267)	<0.001
dysglycaemia	HOMA-IR	Direct	1.070 (1.038, 1.102)	<0.001	1.132 (1.087, 1.178)	<0.001
	TyG	Direct	1.205 (1.162, 1.249)	<0.001	1.275 (1.242, 1.309)	<0.001
	TyG-BMI	Direct	1.196 (1.157, 1.236)	<0.001	1.229 (1.199, 1.261)	<0.001
	TG/HDL-C	None	1.077 (1.048, 1.107)	<0.001	1.069 (1.030, 1.108)	<0.001
	METS-IR	Direct	1.166 (1.128, 1.205)	<0.001	1.235 (1.203, 1.268)	<0.001
elevated triglycerides	HOMA-IR	None	1.095 (1.053, 1.139)	<0.001	1.203 (1.133, 1.278)	<0.001
	TyG	Direct	2.064 (1.938, 2.199)	<0.001	2.436 (2.228, 2.664)	<0.001
	TyG-BMI	Direct	1.654 (1.575, 1.737)	<0.001	1.511 (1.420, 1.608)	<0.001
	TG/HDL-C	Direct	1.270 (1.180, 1.367)	<0.001	1.167 (1.108, 1.228)	<0.001
	METS-IR	Direct	1.587 (1.520, 1.658)	<0.001	1.517 (1.419, 1.623)	<0.001
reduced HDL-C	HOMA-IR	None	1.080 (1.045, 1.117)	<0.001	1.219 (1.167, 1.273)	<0.001
	TyG	None	1.466 (1.399, 1.536)	<0.001	1.588 (1.506, 1.674)	<0.001
	TyG-BMI	None	1.364 (1.301, 1.429)	<0.001	1.407 (1.354, 1.462)	<0.001
	TG/HDL-C	Direct	1.237 (1.157, 1.323)	<0.001	1.119 (1.065, 1.175)	<0.001
	METS-IR	Direct	1.618 (1.545, 1.694)	<0.001	1.539 (1.480, 1.601)	<0.001

Component outcome	Index	Overlap category	CHNS		NHANES	
			PR (95% CI)	P value	PR (95% CI)	P value
elevated waist circumference	HOMA-IR	None	1.010 (0.996, 1.024)	0.162	1.036 (1.023, 1.049)	<0.001
	TyG	None	1.030 (1.013, 1.047)	<0.001	1.054 (1.039, 1.069)	<0.001
	TyG-BMI	Adiposity-related	1.071 (1.053, 1.089)	<0.001	1.078 (1.061, 1.096)	<0.001
	TG/HDL-C	None	1.018 (1.005, 1.030)	0.005	1.025 (1.009, 1.042)	0.004
	METS-IR	Adiposity-related	1.067 (1.050, 1.084)	<0.001	1.077 (1.060, 1.095)	<0.001

Note. Component-specific models used the common BMI-free specification, with community-clustered robust variance in CHNS and survey-weighted estimation in NHANES. Overlap category indicates direct formula-to-component overlap, adiposity-related overlap, or no direct formula overlap. CHNS models used n=1,721 complete cases; NHANES models used n=2,799 complete cases.

Abbreviations: CHNS, China Health and Nutrition Survey; CI, confidence interval; HDL-C, high-density lipoprotein cholesterol; NHANES, National Health and Nutrition Examination Survey; PR, prevalence ratio.

Supplementary Table S6. Alignment between surrogate indices and the composite metabolic dysregulation burden score

Index	CHNS				NHANES			
	Spearman rho	Pearson r	Adjusted beta (95% CI)	P value	Spearman rho	Pearson r	Adjusted beta (95% CI)	P value
HOMA-IR	0.400	0.289	0.138 (0.077, 0.198)	<0.001	0.626	0.386	0.332 (0.251, 0.413)	<0.001
TyG	0.675	0.719	0.377 (0.354, 0.400)	<0.001	0.676	0.721	0.424 (0.392, 0.455)	<0.001
TyG-BMI	0.722	0.730	0.385 (0.362, 0.408)	<0.001	0.747	0.691	0.380 (0.355, 0.404)	<0.001
TG/HDL-C	0.664	0.621	0.331 (0.273, 0.388)	<0.001	0.688	0.624	0.370 (0.273, 0.467)	<0.001
METS-IR	0.759	0.775	0.415 (0.394, 0.437)	<0.001	0.789	0.733	0.405 (0.380, 0.431)	<0.001

Note. The composite burden score is the arithmetic mean of five within-database standardized domains: adiposity (standardised waist circumference), blood pressure (mean of standardised systolic and diastolic blood pressure), glycaemic status (mean of standardised fasting glucose and HbA1c), triglycerides, and reduced HDL-C (negative of standardised HDL-C). Correlations used the frozen eligible populations (CHNS n=1,751; NHANES n=2,953). Adjusted linear models used the common BMI-free covariates and complete cases (CHNS n=1,721; NHANES n=2,799); CHNS standard errors were clustered by community and NHANES estimates incorporated the complex survey design.

Abbreviations: BMI, body mass index; CHNS, China Health and Nutrition Survey; CI, confidence interval; HDL-C, high-density lipoprotein cholesterol; NHANES, National Health and Nutrition Examination Survey; SD, standard deviation.

Supplementary Table S7. Cross-database rank-consistency summary

Index	Spearman rank CHNS/NHANES	Adjusted beta rank CHNS/NHANES	AUC-MUP rank CHNS/NHANES	Maximum rank difference	Direction agreement	Rank consistency status
HOMA-IR	5/5	5/5	5/1	4	YES	Lower (maximum rank difference = 4)
TyG	3/4	3/1	2/3	2	YES	Moderate (maximum rank difference = 2)
TG/HDL-C	4/3	4/4	4/5	1	YES	Higher (maximum rank difference ≤ 1)
TyG-BMI	2/2	2/3	3/4	1	YES	Higher (maximum rank difference ≤ 1)
METS-IR	1/1	1/2	1/2	1	YES	Higher (maximum rank difference ≤ 1)

Note. Ranks are descending within each database; rank 1 indicates the strongest positive signal. Rank consistency summarizes cross-database agreement across correlation with the composite burden, adjusted beta for the composite burden, and AUC for the component-defined MUP. CHNS AUC ranks are empirical; NHANES AUC ranks use survey-weighted AUCs. The labels higher, moderate, and lower describe rank consistency only and do not imply external validation or generalizability.

Abbreviations: AUC, area under the receiver operating characteristic curve; CHNS, China Health and Nutrition Survey; MUP, metabolically unhealthy phenotype; NHANES, National Health and Nutrition Examination Survey.

Supplementary Table S8. Age-stage stratified associations and interaction tests

Index	Age-stage	CHNS			NHANES		
		PR (95% CI)	P value	P interaction	PR (95% CI)	P value	P interaction
HOMA-IR	<45 years	1.219 (1.119, 1.328)	<0.001	<0.001	1.259 (1.175, 1.348)	<0.001	<0.001
	45–54 years	1.156 (1.103, 1.212)	<0.001	—	1.119 (1.059, 1.183)	<0.001	—
	≥55 years	1.011 (1.004, 1.019)	0.003	—	1.036 (1.019, 1.053)	<0.001	—
TyG	<45 years	1.247 (1.181, 1.316)	<0.001	<0.001	1.265 (1.207, 1.327)	<0.001	<0.001
	45–54 years	1.152 (1.109, 1.198)	<0.001	—	1.148 (1.100, 1.198)	<0.001	—
	≥55 years	1.066 (1.046, 1.086)	<0.001	—	1.091 (1.063, 1.121)	<0.001	—
TG/HDL-C	<45 years	1.087 (1.024, 1.154)	0.006	0.003	1.052 (1.011, 1.094)	0.016	0.420
	45–54 years	1.085 (1.055, 1.116)	<0.001	—	1.058 (1.020, 1.096)	0.004	—
	≥55 years	1.039 (1.024, 1.055)	<0.001	—	1.092 (1.059, 1.125)	<0.001	—
TyG-BMI	<45 years	1.252 (1.190, 1.317)	<0.001	<0.001	1.236 (1.189, 1.285)	<0.001	<0.001
	45–54 years	1.152 (1.110, 1.196)	<0.001	—	1.134 (1.088, 1.181)	<0.001	—
	≥55 years	1.058 (1.039, 1.078)	<0.001	—	1.070 (1.043, 1.098)	<0.001	—
METS-IR	<45 years	1.253 (1.188, 1.321)	<0.001	<0.001	1.253 (1.207, 1.301)	<0.001	<0.001
	45–54 years	1.155 (1.113, 1.198)	<0.001	—	1.140 (1.091, 1.191)	<0.001	—
	≥55 years	1.062 (1.041, 1.083)	<0.001	—	1.077 (1.049, 1.107)	<0.001	—

Note. P interaction was estimated for the interaction between each standardized index and the three-level age-stage variable and is shown once per index block. Age-stage (<45, 45–54, and ≥55 years) is a prespecified proxy and is not interpreted as measured menopausal status. Models used the common BMI-free covariate specification.

Abbreviations: CHNS, China Health and Nutrition Survey; CI, confidence interval; NHANES, National Health and Nutrition Examination Survey; PR, prevalence ratio.

Supplementary Table S9. Full BMI-spectrum sensitivity analyses

Index	BMI category	CHNS			NHANES		
		PR (95% CI)	P value	P interaction	PR (95% CI)	P value	P interaction
HOMA-IR	Overall	1.054 (1.031, 1.078)	<0.001	<0.001	1.122 (1.085, 1.161)	<0.001	0.002
	Normal	1.063 (1.034, 1.093)	<0.001	—	1.160 (1.065, 1.263)	0.001	—
	Overweight	1.038 (1.011, 1.065)	0.006	—	1.103 (1.035, 1.175)	0.004	—
	Obesity	1.007 (0.996, 1.018)	0.184	—	1.047 (1.029, 1.066)	<0.001	—
TyG	Overall	1.350 (1.313, 1.388)	<0.001	<0.001	1.325 (1.290, 1.361)	<0.001	<0.001
	Normal	1.563 (1.493, 1.636)	<0.001	—	1.660 (1.497, 1.842)	<0.001	—
	Overweight	1.162 (1.132, 1.192)	<0.001	—	1.234 (1.187, 1.283)	<0.001	—
	Obesity	1.044 (1.024, 1.064)	<0.001	—	1.106 (1.083, 1.130)	<0.001	—
TG/HDL-C	Overall	1.144 (1.107, 1.183)	<0.001	<0.001	1.080 (1.039, 1.122)	<0.001	<0.001
	Normal	1.243 (1.134, 1.362)	<0.001	—	1.878 (1.618, 2.180)	<0.001	—
	Overweight	1.068 (1.041, 1.096)	<0.001	—	1.089 (1.039, 1.142)	<0.001	—
	Obesity	1.023 (1.012, 1.034)	<0.001	—	1.035 (1.009, 1.061)	0.011	—
TyG-BMI	Overall	1.383 (1.348, 1.419)	<0.001	<0.001	1.371 (1.331, 1.411)	<0.001	<0.001
	Normal	2.338 (2.162, 2.528)	<0.001	—	6.349 (4.318, 9.336)	<0.001	—
	Overweight	1.301 (1.243, 1.363)	<0.001	—	2.048 (1.804, 2.324)	<0.001	—
	Obesity	1.051 (1.026, 1.078)	<0.001	—	1.113 (1.087, 1.139)	<0.001	—
METS-IR	Overall	1.386 (1.351, 1.423)	<0.001	<0.001	1.380 (1.339, 1.422)	<0.001	<0.001
	Normal	1.939 (1.653, 2.276)	<0.001	—	5.787 (3.903, 8.581)	<0.001	—
	Overweight	1.256 (1.205, 1.309)	<0.001	—	1.871 (1.686, 2.077)	<0.001	—
	Obesity	1.058 (1.032, 1.084)	<0.001	—	1.127 (1.100, 1.154)	<0.001	—

Note. Full BMI-spectrum sensitivity analyses included normal weight, overweight, and obesity and excluded underweight women. CHNS cut points were 18.5/24/28 kg/m² and NHANES cut points were 18.5/25/30 kg/m². P interaction was estimated for each index-by-BMI-category term and is shown once per index block. TyG-BMI and METS-IR results are exploratory scale/construct sensitivity analyses because BMI is embedded in these indices.

Abbreviations: BMI, body mass index; CHNS, China Health and Nutrition Survey; CI, confidence interval; NHANES, National Health and Nutrition Examination Survey; PR, prevalence ratio.

Supplementary Table S10. Sensitivity analyses using alternative phenotype definitions

Outcome definition	Index	CHNS		NHANES	
		PR (95% CI)	P value	PR (95% CI)	P value
MUP ≥3 abnormal components	HOMA-IR	1.051 (1.022, 1.081)	<0.001	1.115 (1.078, 1.154)	<0.001
	TyG	1.395 (1.346, 1.446)	<0.001	1.415 (1.362, 1.471)	<0.001
	TG/HDL-C	1.162 (1.111, 1.216)	<0.001	1.102 (1.053, 1.154)	<0.001
	TyG-BMI	1.358 (1.311, 1.406)	<0.001	1.373 (1.325, 1.424)	<0.001
	METS-IR	1.370 (1.326, 1.415)	<0.001	1.408 (1.356, 1.462)	<0.001
Alternative waist-cutoff phenotype	HOMA-IR	1.026 (1.007, 1.044)	0.006	1.048 (1.029, 1.067)	<0.001
	TyG	1.171 (1.143, 1.199)	<0.001	1.132 (1.111, 1.153)	<0.001
	TG/HDL-C	1.086 (1.059, 1.114)	<0.001	1.050 (1.019, 1.081)	0.002
	TyG-BMI	1.189 (1.163, 1.216)	<0.001	1.130 (1.110, 1.149)	<0.001
	METS-IR	1.195 (1.169, 1.222)	<0.001	1.143 (1.122, 1.165)	<0.001

Note. Sensitivity analyses used MUP defined as at least three abnormal components and an audited alternative waist-circumference cutoff of 85 cm. CHNS and NHANES results were regenerated from the frozen analysis populations with the database-specific complex-variance procedures.

Abbreviations: CHNS, China Health and Nutrition Survey; CI, confidence interval; MUP, metabolically unhealthy phenotype; NHANES, National Health and Nutrition Examination Survey; PR, prevalence ratio.

Supplementary Table S11. Exploratory ROC/AUC details for the primary phenotype and blood pressure abnormality

Outcome	Index	CHNS				NHANES			
		AUC (95% CI)	Youden cutoff	Sensitivity	Specificity	AUC (95% CI)	Youden cutoff	Sensitivity	Specificity
Component-defined MUP	HOMA-IR	0.690 (0.658, 0.722)	2.96	0.538	0.756	0.789 (0.766, 0.812); empirical 0.796	2.61	0.682	0.785
	TyG	0.814 (0.791, 0.837)	8.71	0.616	0.904	0.794 (0.770, 0.815); empirical 0.792	8.46	0.644	0.800
	TG/HDL-C	0.806 (0.782, 0.829)	1.07	0.596	0.896	0.777 (0.754, 0.797); empirical 0.766	0.66	0.674	0.745
	TyG-BMI	0.810 (0.784, 0.836)	220.9	0.743	0.756	0.774 (0.747, 0.800); empirical 0.789	258.3	0.667	0.760
	METS-IR	0.823 (0.799, 0.847)	37.6	0.693	0.828	0.780 (0.751, 0.805); empirical 0.794	41.6	0.753	0.699
BP abnormality	HOMA-IR	0.593 (0.566, 0.619)	3.43	0.473	0.689	0.600 (0.580, 0.621); empirical	2.61	0.679	0.484
	TyG	0.619 (0.593, 0.645)	8.92	0.508	0.684	0.609 (0.589, 0.630); empirical	8.50	0.621	0.548
	TG/HDL-C	0.583 (0.557, 0.610)	1.44	0.435	0.702	0.556 (0.535, 0.577); empirical	0.69	0.627	0.476
	TyG-BMI	0.649 (0.623, 0.675)	238.3	0.528	0.711	0.619 (0.599, 0.639); empirical	247.3	0.757	0.417
	METS-IR	0.618 (0.592, 0.644)	41.7	0.429	0.770	0.591 (0.570, 0.611); empirical	50.8	0.410	0.729

Note. CHNS AUCs and all Youden cutoffs, sensitivities, and specificities are empirical and internally derived. For the primary MUP outcome, the first NHANES AUC is survey-weighted (WTSAF8YR, SDMVSTRA, and SDMVPSU) and the empirical AUC follows after the semicolon; BP-outcome NHANES AUCs are empirical because a locked survey-weighted BP AUC was not available. Cutoffs should be interpreted as exploratory internal thresholds, not validated clinical cut points. Survey-weighted pairwise DeLong tests were not performed. The corresponding survey-weighted NHANES ROC curves are shown in Supplementary Figure S7.

Abbreviations: AUC, area under the receiver operating characteristic curve; BP, blood pressure; CHNS, China Health and Nutrition Survey; MUP, metabolically unhealthy phenotype; NHANES, National Health and Nutrition Examination Survey; ROC, receiver operating characteristic.

Supplementary Table S12. Sensitivity analysis excluding known pregnancy in CHNS

Index	Original CHNS		Preferred CHNS		Relative change, %
	Original eligible CHNS (n=1,771; complete-case n=1,741)	P value	Preferred eligible CHNS (n=1,751; complete-case n=1,721)	P value	
HOMA-IR	1.021 (1.006, 1.036)	0.004	1.020 (1.006, 1.034)	0.006	-0.09
TyG	1.125 (1.103, 1.148)	<0.001	1.123 (1.101, 1.146)	<0.001	-0.20
TG/HDL-C	1.064 (1.043, 1.086)	<0.001	1.064 (1.043, 1.085)	<0.001	-0.01
TyG-BMI	1.124 (1.103, 1.145)	<0.001	1.123 (1.102, 1.144)	<0.001	-0.06
METS-IR	1.130 (1.109, 1.151)	<0.001	1.131 (1.110, 1.153)	<0.001	0.12

Note. The preferred eligible CHNS population excluded 20 known pregnancies (U56=1) while retaining missing pregnancy status as unknown. The primary regression further excluded 30 participants with missing D3KCAL/D3KCAL_z; no imputation was performed. Therefore, eligible and complete-case sample sizes are shown separately. Relative change compares the adjusted PRs after the pregnancy exclusion with the corresponding pre-exclusion complete-case analysis.
Abbreviations: CHNS, China Health and Nutrition Survey; CI, confidence interval; PR, prevalence ratio.

Supplementary Table S13. Strict non-overlap associations after excluding formula-overlapping components

Index	Overlapping components removed	Residual-component outcome	CHNS			NHANES		
		Strict residual outcome (≥2 remaining abnormal components)	PR (95% CI)	P value	No. of events	PR (95% CI)	P value	No. of events
HOMA-IR	FG	BP+TG+HDL+WC; ≥2 abnormal	1.038 (1.018, 1.059)	<0.001	1301	1.105 (1.072, 1.140)	<0.001	2028
TyG	FG+TG	BP+HDL+WC; ≥2 abnormal	1.173 (1.139, 1.209)	<0.001	1174	1.226 (1.194, 1.259)	<0.001	1969
TyG-BMI	FG+TG+WC	BP+HDL; ≥2 abnormal	1.576 (1.462, 1.700)	<0.001	406	1.682 (1.577, 1.793)	<0.001	588
TG/HDL-C	TG+HDL	BP+FG+WC; ≥2 abnormal	1.060 (1.043, 1.078)	<0.001	1251	1.062 (1.028, 1.098)	<0.001	2131
METS-IR	FG+TG+HDL+WC	BP only; a ≥2-component residual outcome cannot be defined	Not estimable	—	—	Not estimable	—	—

Note. For each index, all metabolic components entering its formula were removed and the strict residual outcome required at least two of the remaining components to be abnormal. Models used the common BMI-free specification. For METS-IR, only blood pressure remained after removing glycaemia, triglycerides, HDL-C, and adiposity; therefore a ≥2-component residual outcome was not estimable. The separate BP-only endpoint is reported in Supplementary Table S4 and is not presented as a strict residual-MUP result.

Abbreviations: BP, blood pressure; CHNS, China Health and Nutrition Survey; CI, confidence interval; FG, fasting glucose; HDL, reduced high-density lipoprotein cholesterol; NHANES, National Health and Nutrition Examination Survey; PR, prevalence ratio; TG, elevated triglycerides; WC, elevated waist circumference.

Supplementary Table S14. Robustness of associations to the functional form of the surrogate indices

Index	CHNS			NHANES		
	Log-transformed (per 1-SD)	IQR-scaled (per 1-IQR)	Winsorized (per 1-SD)	Log-transformed (per 1-SD)	IQR-scaled (per 1-IQR)	Winsorized (per 1-SD)
HOMA-IR	1.073 (1.052, 1.093)	1.010 (1.004, 1.016)	1.048 (1.037, 1.060)	1.179 (1.150, 1.209)	1.037 (1.025, 1.050)	1.107 (1.090, 1.124)
TyG	1.139 (1.115, 1.164)	1.179 (1.147, 1.211)	1.135 (1.111, 1.158)	1.177 (1.152, 1.204)	1.227 (1.194, 1.260)	1.174 (1.149, 1.200)
TyG-BMI	1.134 (1.111, 1.157)	1.156 (1.129, 1.183)	1.126 (1.105, 1.147)	1.171 (1.148, 1.195)	1.194 (1.166, 1.223)	1.155 (1.134, 1.176)
TG/HDL-C	1.138 (1.114, 1.162)	1.046 (1.031, 1.061)	1.087 (1.071, 1.104)	1.177 (1.150, 1.204)	1.034 (1.014, 1.055)	1.124 (1.106, 1.143)
METS-IR	1.144 (1.120, 1.167)	1.166 (1.139, 1.193)	1.134 (1.113, 1.156)	1.186 (1.161, 1.212)	1.216 (1.185, 1.248)	1.169 (1.145, 1.193)

Note. Each cell is the prevalence ratio (95% CI) under an alternative index treatment: natural-log transformation followed by standardisation, with the model refitted using the log-transformed, standardised exposure; an interquartile-range (IQR)-scaled estimate obtained by rescaling the per-1-SD model coefficient and standard error by the ratio of the index's IQR to its SD, without refitting the model (this rescaling changes the point estimate and CI but leaves the Wald test statistic and P value identical to the per-1-SD model); or winsorisation of the exposure at the 1st and 99th percentiles followed by standardisation, with the model refitted using the winsorised, standardised exposure. Models used the common BMI-free covariate specification. CHNS values were updated from the locked n=1,721 complete-case rerun; NHANES values used the verified n=2,799 survey-weighted analysis (WTSAF8YR, SDMVSTRA, SDMVPSU).

Abbreviations: CHNS, China Health and Nutrition Survey; CI, confidence interval; IQR, interquartile range; NHANES, National Health and Nutrition Examination Survey; PR, prevalence ratio; SD, standard deviation.

Supplementary Table S15. Associations with a harmonized metabolically unhealthy phenotype (uniform waist cut-off, medication excluded)

Index	Primary harmonized phenotype (waist ≥ 85 cm, no medication)				Sensitivity: uniform BMI ≥ 25 kg/m²	
	CHNS PR (95% CI)	P value	NHANES PR (95% CI)	P value	CHNS PR (95% CI)	NHANES PR (95% CI)
HOMA-IR	1.036 (1.016, 1.057)	<0.001	1.074 (1.049, 1.099)	<0.001	1.033 (1.012, 1.054)	1.074 (1.049, 1.099)
TyG	1.189 (1.161, 1.219)	<0.001	1.150 (1.128, 1.172)	<0.001	1.155 (1.128, 1.183)	1.150 (1.128, 1.172)
TyG-BMI	1.189 (1.163, 1.216)	<0.001	1.130 (1.110, 1.149)	<0.001	1.156 (1.129, 1.184)	1.130 (1.110, 1.149)
TG/HDL-C	1.095 (1.065, 1.125)	<0.001	1.054 (1.022, 1.088)	0.002	1.075 (1.047, 1.104)	1.054 (1.022, 1.088)
METS-IR	1.195 (1.169, 1.222)	<0.001	1.143 (1.122, 1.165)	<0.001	1.159 (1.133, 1.186)	1.143 (1.122, 1.165)

Note. The harmonized phenotype used an identical waist-circumference cutoff of 85 cm in both databases and excluded medication-based components. The BMI ≥25 kg/m² analysis is shown as an additional harmonization sensitivity. Estimates use the common BMI-free covariate specification and the database-appropriate clustered or survey variance estimator.

Abbreviations: BMI, body mass index; CHNS, China Health and Nutrition Survey; CI, confidence interval; NHANES, National Health and Nutrition Examination Survey; PR, prevalence ratio; SD, standard deviation.

Supplementary Table S16. Associations after excluding participants with diabetes

Index	CHNS			NHANES		
	PR (95% CI)	P value	n	PR (95% CI)	P value	n
HOMA-IR	1.142 (1.093, 1.192)	<0.001	1473	1.409 (1.311, 1.515)	<0.001	2153
TyG	1.173 (1.140, 1.207)	<0.001	1473	1.247 (1.203, 1.293)	<0.001	2153
TyG-BMI	1.161 (1.133, 1.191)	<0.001	1473	1.183 (1.153, 1.214)	<0.001	2153
TG/HDL-C	1.101 (1.064, 1.140)	<0.001	1473	1.128 (1.065, 1.195)	<0.001	2153
METS-IR	1.171 (1.142, 1.201)	<0.001	1473	1.207 (1.173, 1.242)	<0.001	2153

Note. Participants with measured or treated diabetes were excluded, leaving 1,473 CHNS and 2,153 NHANES participants in the complete-case models. Estimates are prevalence ratios per 1-SD increment under the common BMI-free specification, with community-clustered robust variance in CHNS and survey-weighted estimation in NHANES.

Abbreviations: CHNS, China Health and Nutrition Survey; CI, confidence interval; NHANES, National Health and Nutrition Examination Survey; PR, prevalence ratio; SD, standard deviation.

Supplementary Table S17. Associations with the number of metabolic abnormalities modelled as a count outcome

Index	CHNS		NHANES	
	Count ratio (95% CI)	P value	Count ratio (95% CI)	P value
HOMA-IR	1.055 (1.030, 1.080)	<0.001	1.124 (1.086, 1.163)	<0.001
TyG	1.283 (1.261, 1.305)	<0.001	1.314 (1.292, 1.337)	<0.001
TyG-BMI	1.243 (1.220, 1.267)	<0.001	1.223 (1.202, 1.244)	<0.001
TG/HDL-C	1.142 (1.099, 1.186)	<0.001	1.089 (1.043, 1.138)	<0.001
METS-IR	1.264 (1.242, 1.286)	<0.001	1.247 (1.224, 1.269)	<0.001

Note. The outcome was the count of abnormal metabolic components (0–5). Count ratios per 1-SD increment were estimated under the common BMI-free specification using modified-Poisson models with community-clustered robust variance in CHNS and survey-weighted estimation in NHANES.

Abbreviations: CHNS, China Health and Nutrition Survey; CI, confidence interval; NHANES, National Health and Nutrition Examination Survey; SD, standard deviation.

Supplementary Table S18. E-values for the associations of surrogate indices with the metabolically unhealthy phenotype

Index	Per 1-SD increment				Q4 vs Q1			
	CHNS		NHANES		CHNS		NHANES	
	Point	CI limit	Point	CI limit	Point	CI limit	Point	CI limit
HOMA-IR	1.19	1.11	1.38	1.29	1.77	1.60	2.62	2.34
TyG	1.52	1.46	1.61	1.55	2.19	1.97	2.58	2.33
TyG-BMI	1.49	1.44	1.57	1.51	2.32	2.09	2.50	2.26
TG/HDL-C	1.34	1.27	1.31	1.18	2.28	2.06	2.59	2.30
METS-IR	1.52	1.46	1.60	1.54	2.44	2.20	2.68	2.43

Note. E-values quantify the minimum strength of association, on the risk-ratio scale, that an unmeasured confounder would need with both exposure and outcome to fully explain the observed association. Point E-values were computed from the prevalence ratios in Table 2 (BMI-free comparable specification), and CI-limit E-values from the confidence bound nearest the null. E-values were consistently above 1.0 and largest for the quartile contrasts.

Abbreviations: CHNS, China Health and Nutrition Survey; CI, confidence interval; NHANES, National Health and Nutrition Examination Survey; Q, quartile; SD, standard deviation.

Supplementary Table S19. Alignment of surrogate indices with the composite burden score before and after removing formula-overlapping domains

Index	Overlapping domains removed	CHNS		NHANES	
		Full burden rho	Domain-excluded rho	Full burden rho	Domain-excluded rho
HOMA-IR	glycaemia	0.400	0.316	0.626	0.559
TyG	glycaemia+triglycerides	0.675	0.399	0.676	0.418
TyG-BMI	glycaemia+triglycerides+adiposity	0.722	0.585	0.747	0.455
TG/HDL-C	triglycerides+HDL-C	0.664	0.245	0.688	0.279
METS-IR	glycaemia+triglycerides+HDL-C+adiposity	0.759	0.219	0.789	0.127

Note. Spearman correlations were computed between each index and the composite burden before and after removing all formula-overlapping domains. The full burden comprised adiposity, blood pressure, glycaemia, triglycerides, and reduced HDL-C. Domain-excluded correlations isolate non-overlapping information and are interpreted descriptively as mathematical/domain concordance, not as independent validation.

Abbreviations: CHNS, China Health and Nutrition Survey; HDL-C, high-density lipoprotein cholesterol; METS-IR, metabolic score for insulin resistance; NHANES, National Health and Nutrition Examination Survey; TyG, triglyceride-glucose index; TyG-BMI, triglyceride-glucose body mass index.

Supplementary Table S20. Comparison of model-based (naïve, i.i.d.) and community-clustered robust standard errors for the CHNS analyses (primary, BMI-excluded models).

Panel A. Per 1-standard-deviation increase in each surrogate index (adjusted log-Poisson regression for the metabolic-unhealthy phenotype).

Surrogate index	PR	SE, naïve	SE, cluster-robust	95% CI, naïve	95% CI, cluster-robust	P value
HOMA-IR	1.027	0.0215	0.0079	(0.984, 1.071)	(1.011, 1.043)	<0.001
TyG	1.133	0.0259	0.0105	(1.077, 1.192)	(1.110, 1.157)	<0.001
TyG-BMI	1.123	0.0251	0.0095	(1.069, 1.180)	(1.102, 1.144)	<0.001
TG/HDL-C	1.070	0.0213	0.0108	(1.026, 1.116)	(1.047, 1.093)	<0.001
METS-IR	1.131	0.0245	0.0096	(1.078, 1.187)	(1.110, 1.153)	<0.001

Panel B. Highest vs lowest quartile (Q4 vs Q1) of each surrogate index (adjusted log-Poisson regression).

Surrogate index	PR	SE, naïve	SE, cluster-robust	95% CI, naïve	95% CI, cluster-robust	P value
HOMA-IR	1.234	0.0749	0.0296	(1.066, 1.429)	(1.165, 1.308)	<0.001
TyG	1.418	0.0781	0.0359	(1.217, 1.652)	(1.322, 1.521)	<0.001
TyG-BMI	1.478	0.0787	0.0375	(1.267, 1.724)	(1.373, 1.590)	<0.001
TG/HDL-C	1.458	0.0770	0.0362	(1.254, 1.696)	(1.359, 1.566)	<0.001
METS-IR	1.537	0.0776	0.0390	(1.320, 1.789)	(1.423, 1.659)	<0.001

Note: The community-clustered robust standard errors (HC0, clustered by COMMID) were used for all CHNS effect estimates reported in the main and supplementary association tables. The cluster-robust confidence intervals and P values reproduce the locked CHNS primary results. The complete-case analysis included n=1,721 participants, 1,473 MUP events, and 217 communities. Point estimates are identical under the two variance estimators; only standard errors and confidence intervals differ.

Supplementary Table S21. Nonlinearity of index–outcome associations: restricted cubic spline Wald tests

Index	CHNS（n）	CHNS<br>Nonlinear Wald (df=2)	CHNS<br>P value	NHANES<br>n	NHANES<br>Nonlinear Wald (df=2)	NHANES<br>P value
HOMA-IR	1721	70.69	<0.001	2799	142.04	<0.001
TyG	1721	89.47	<0.001	2799	83.57	<0.001
TyG-BMI	1721	100.40	<0.001	2799	66.02	<0.001
TG/HDL-C	1721	133.22	<0.001	2799	165.53	<0.001
METS-IR	1721	104.73	<0.001	2799	77.30	<0.001

Note: Restricted cubic splines with four knots (Harrell default percentiles) were fitted for each index on its raw scale, using the common BMI-free covariate specification (CHNS: HC0 standard errors clustered by COMMID; NHANES: survey-weighted quasi-Poisson regression incorporating WTSAF8YR, SDMVSTRA and SDMVPSU). The nonlinear Wald test jointly tests the two non-linear spline coefficients against the null of a purely linear association (2 degrees of freedom); a significant test indicates that the association departs from linearity on the per-1-SD scale used elsewhere in the manuscript. Corresponding prevalence-ratio curves, expressed relative to the database-specific median index value, are shown in Supplementary Figure S8.

Abbreviations: CHNS, China Health and Nutrition Survey; HOMA-IR, homeostasis model assessment of insulin resistance; METS-IR, metabolic score for insulin resistance; NHANES, National Health and Nutrition Examination Survey; TyG, triglyceride-glucose index; TyG-BMI, triglyceride-glucose body mass index.

Supplementary Figure S1. Associations across four sequential adjustment models.

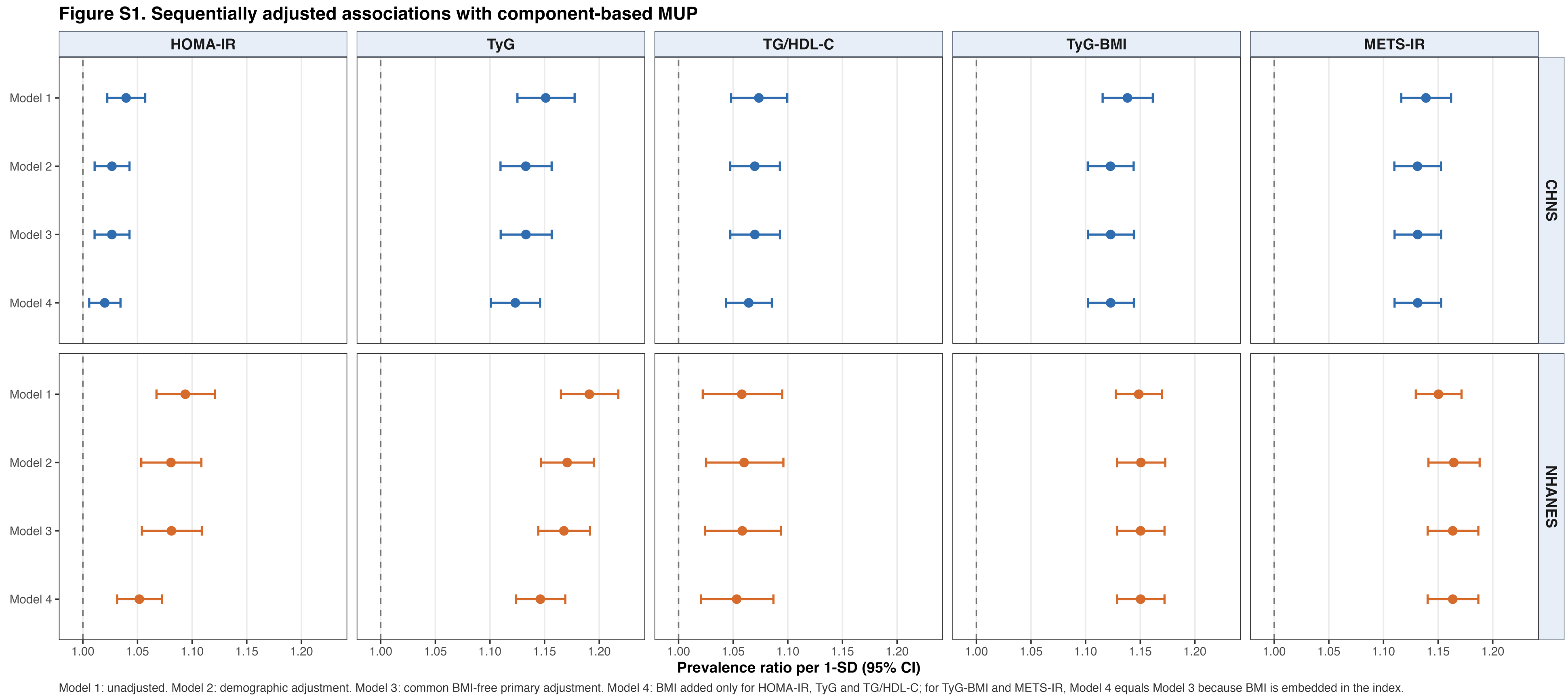

Points are prevalence ratios per 1-SD increment and horizontal bars are 95% confidence intervals. Model 1 was unadjusted. Model 2 added age and urban/rural residence and education (CHNS) or age, race/ethnicity and education (NHANES). Model 3, the common, BMI-free, primary comparative model for all five indices, additionally added alcohol drinking and total energy intake (CHNS) or smoking and total energy intake (NHANES). Model 4, a secondary sensitivity model for HOMA-IR, TyG and TG/HDL-C only, additionally added BMI; BMI is not added for TyG-BMI or METS-IR in either model because it is embedded in those index formulas. CHNS analyses used n=1,721 complete cases with community-clustered robust variance; NHANES analyses used n=2,799 complete cases and the fasting-subsample survey design. MUP, metabolically unhealthy phenotype; PR, prevalence ratio; SD, standard deviation.

Supplementary Figure S2. ROC analyses for alternative phenotype definitions in CHNS.

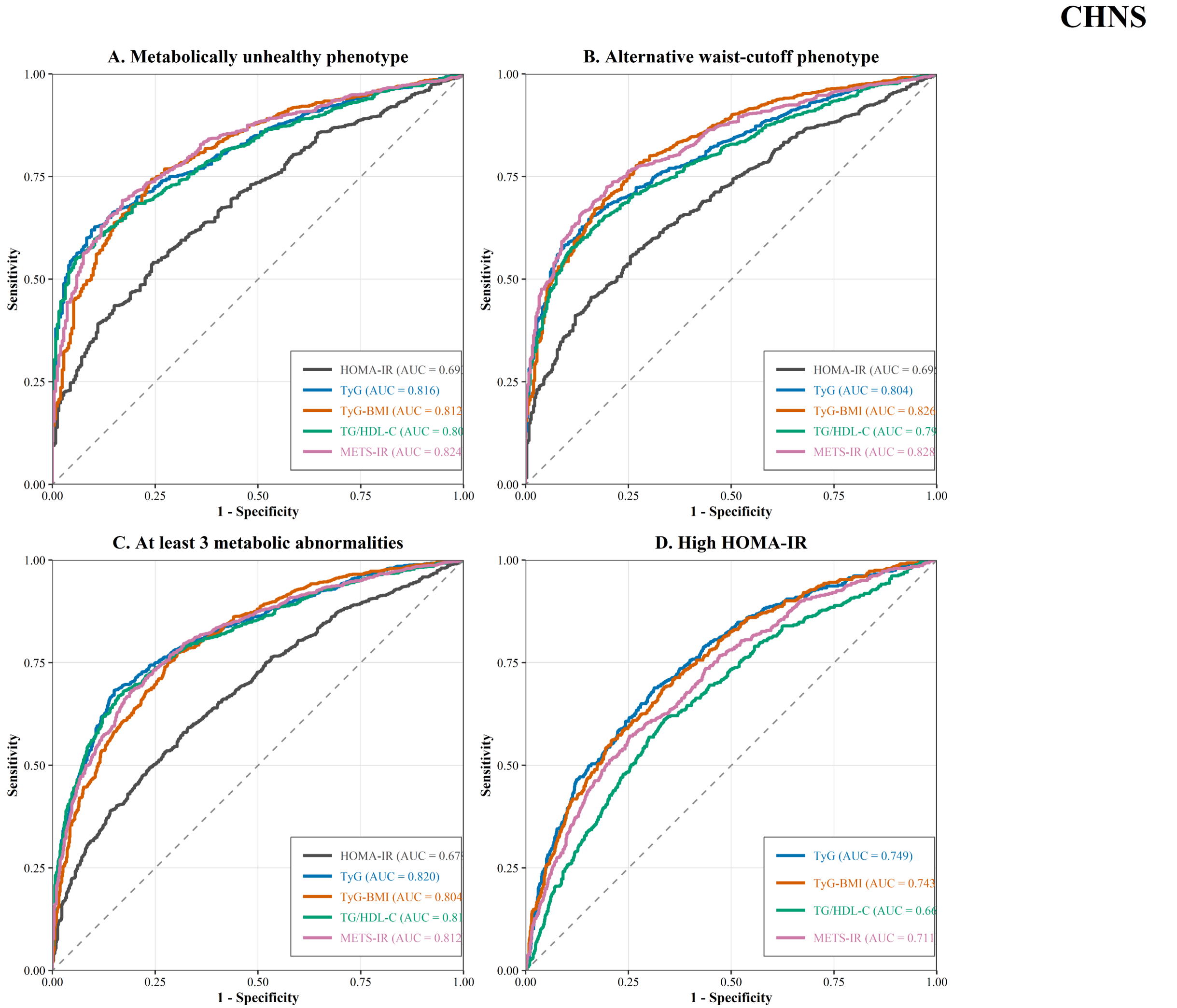

Panels show empirical ROC curves in CHNS across four outcomes: component-defined metabolically unhealthy phenotype, alternative waist-cutoff phenotype, at least three metabolic abnormalities and high HOMA-IR. ROC analyses were conducted in the CHNS final analytic sample of 1,751 participants. Curves describe exploratory internal discrimination and should not be interpreted as validation of clinical diagnostic thresholds. ROC, receiver operating characteristic; AUC, area under the receiver operating characteristic curve; CHNS, China Health and Nutrition Survey.

Supplementary Figure S3. ROC analyses for alternative phenotype definitions in NHANES.

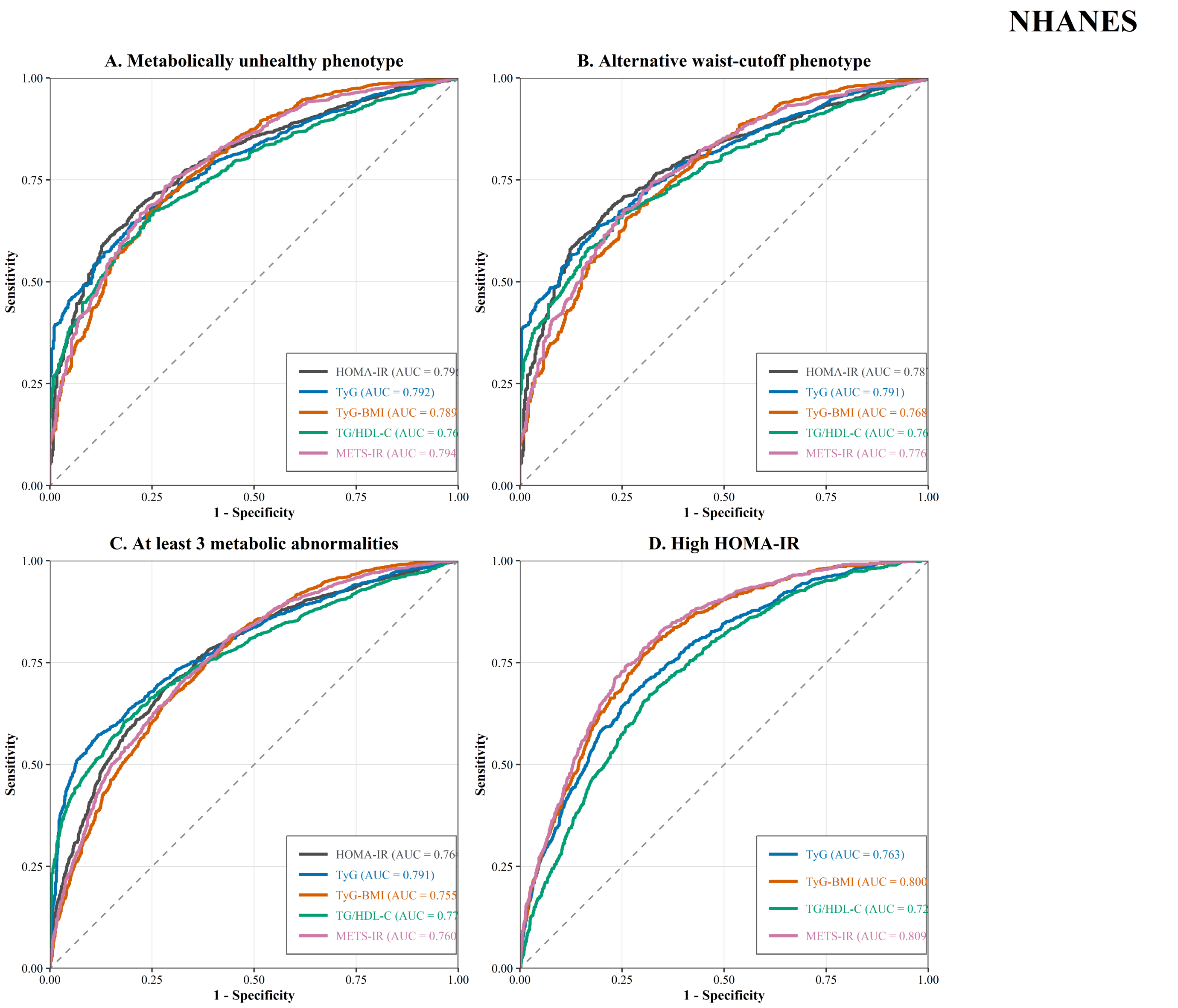

Panels show empirical ROC curves in NHANES across four outcomes: component-defined metabolically unhealthy phenotype, alternative waist-cutoff phenotype, at least three metabolic abnormalities and high HOMA-IR. ROC analyses were conducted in the NHANES final analytic sample of 2,953 participants. Curves describe exploratory internal discrimination and should not be interpreted as validation of clinical diagnostic thresholds. ROC, receiver operating characteristic; AUC, area under the receiver operating characteristic curve; NHANES, National Health and Nutrition Examination Survey.

Supplementary Figure S4. Age-stage and BMI-category subgroup analyses.

Figure S4. Subgroup analyses of insulin-resistance surrogate indices

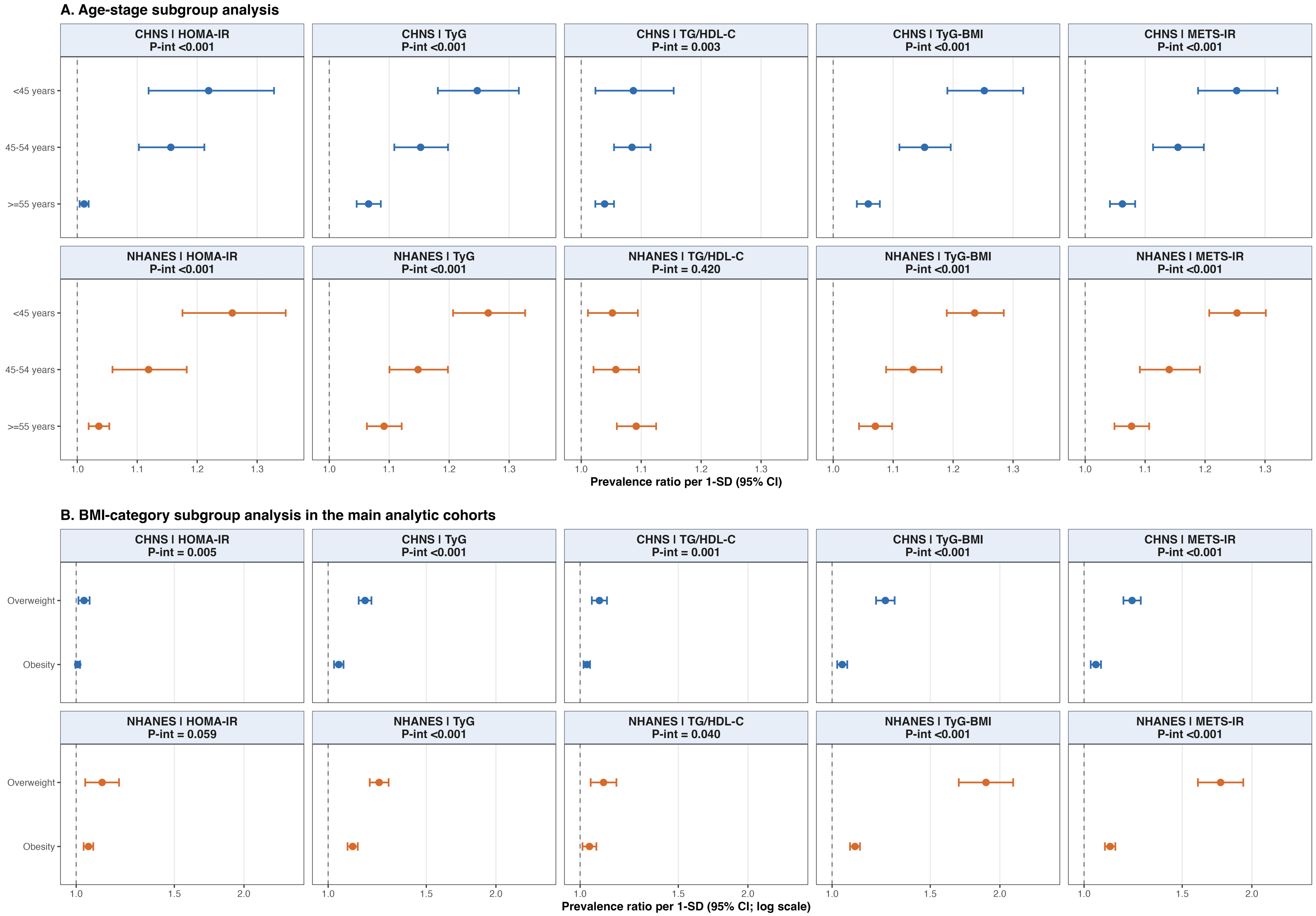

CHNS BMI categories: 24.0–27.9 kg/m² (overweight) and ≥28.0 kg/m² (obesity); NHANES: 25.0–29.9 and ≥30.0 kg/m².
P-interaction values are design-appropriate Wald tests.
BMI-stratified TyG-BMI and METS-IR results are exploratory scale/construct sensitivity analyses because BMI is embedded in these indices.

Panel A shows age-stage results (<45, 45–54, and ≥55 years) for CHNS and NHANES. Panel B shows BMI-category results within the main analytic cohorts: CHNS overweight 24.0–27.9 kg/m² and obesity ≥28.0 kg/m²; NHANES overweight 25.0–29.9 kg/m² and obesity ≥30.0 kg/m². Interaction P values are design-appropriate Wald tests. Age-stage is a proxy, not measured menopausal status. BMI-stratified TyG-BMI and METS-IR results are exploratory because BMI is embedded in those indices. CI, confidence interval; PR, prevalence ratio; SD, standard deviation.

Supplementary Figure S5. Distribution of surrogate indices according to metabolic abnormality burden.

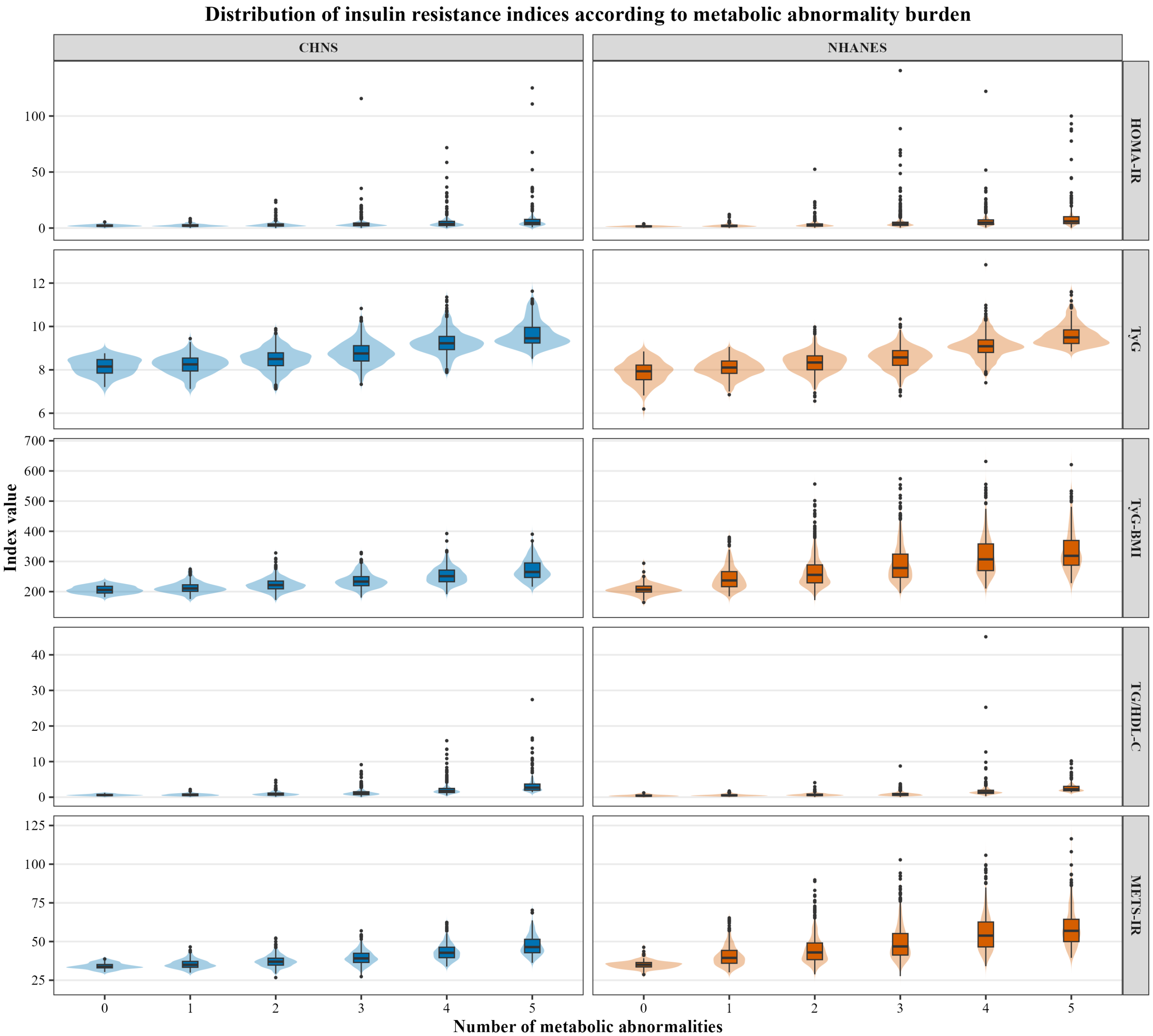

Violin plots show the distribution of each surrogate index according to the number of metabolic abnormalities in CHNS and NHANES. Embedded box plots indicate the median and interquartile range. The figure illustrates graded changes in surrogate index values across increasing metabolic abnormality burden. Distributions were plotted in the final analytic samples: CHNS n = 1,751 and NHANES n = 2,953. CHNS, China Health and Nutrition Survey; NHANES, National Health and Nutrition Examination Survey.

Supplementary Figure S6. Standardized differences in baseline characteristics by metabolic phenotype.

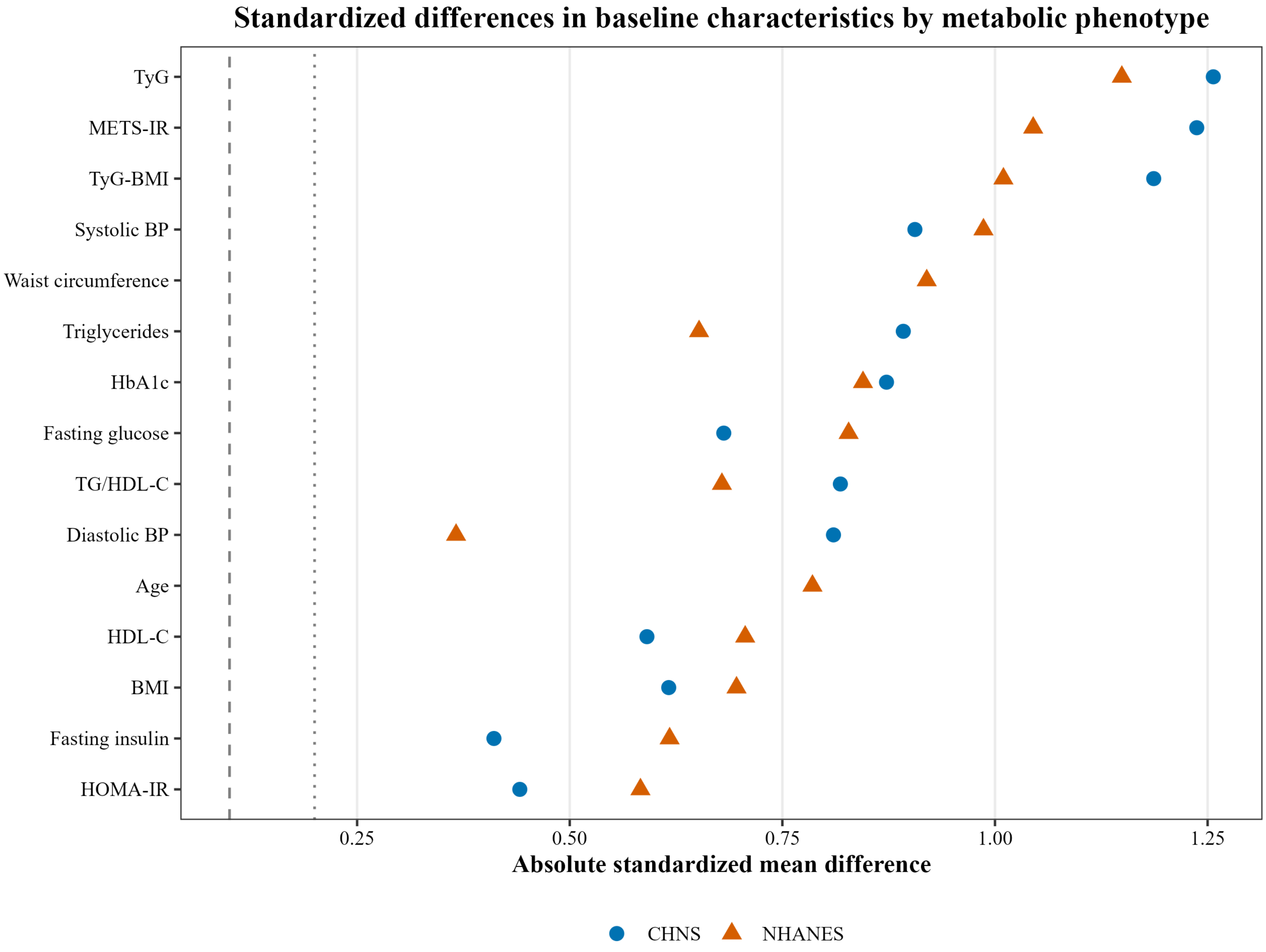

The figure shows absolute standardized mean differences between metabolically healthy and metabolically unhealthy phenotype groups in CHNS and NHANES. Larger values indicate greater separation between phenotype groups. Vertical reference lines indicate commonly used standardized difference thresholds of 0.10 and 0.20. Standardized differences were calculated in the final analytic samples: CHNS n = 1,751 and NHANES n = 2,953. CHNS, China Health and Nutrition Survey; NHANES, National Health and Nutrition Examination Survey; BP, blood pressure; BMI, body mass index.

Supplementary Figure S7. Survey-design-aware NHANES receiver operating characteristic curves for component-defined metabolically unhealthy phenotype.

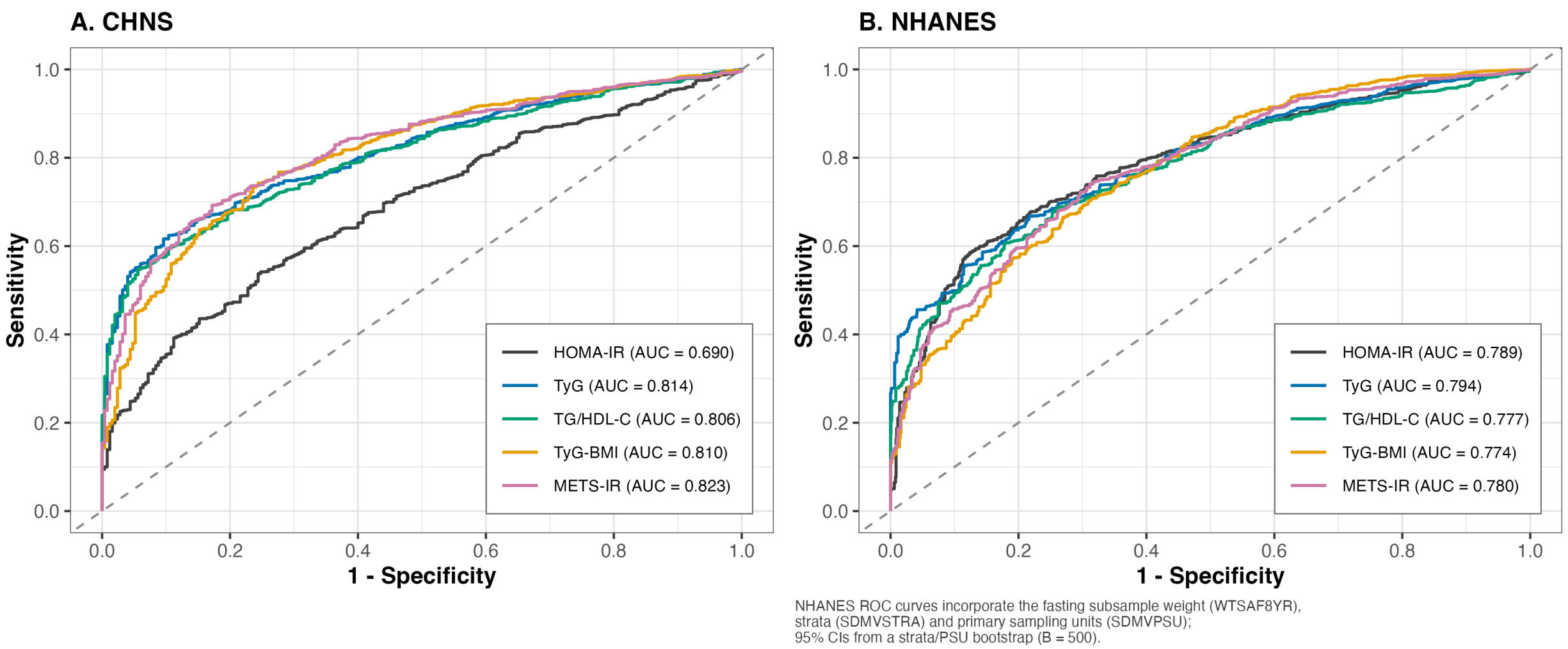

Panel A shows the empirical (unweighted) receiver operating characteristic curve for CHNS, identical to Figure 3A. Panel B shows the NHANES receiver operating characteristic curve incorporating the fasting subsample weight (WTSAF8YR), strata (SDMVSTRA) and primary sampling units (SDMVPSU); 95% confidence intervals for the survey-weighted areas under the curve were obtained from a stratum/PSU cluster bootstrap (B=500). This survey-design-aware analysis complements the unweighted empirical analysis reported as the primary result in Figure 3 and Table 3, and corresponds to the weighted areas under the curve reported in Supplementary Table S11. CHNS, China Health and Nutrition Survey; NHANES, National Health and Nutrition Examination Survey; ROC, receiver operating characteristic; AUC, area under the receiver operating characteristic curve; PSU, primary sampling unit.

Supplementary Figure S8. Restricted cubic spline associations between surrogate indices and component-defined metabolically unhealthy phenotype.

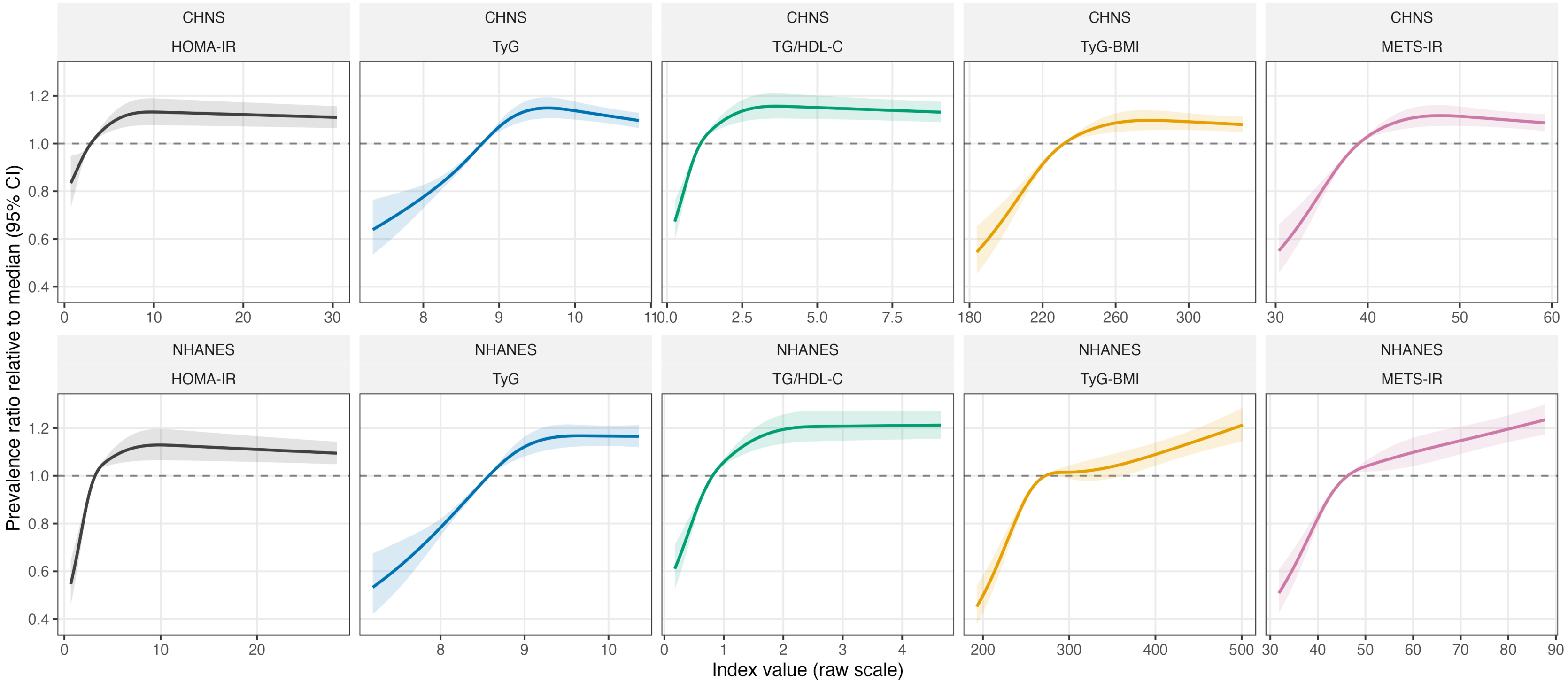

Curves show restricted cubic spline (four knots) prevalence ratios for component-defined metabolically unhealthy phenotype across the raw range (1st–99th percentile) of each surrogate index, expressed relative to the database-specific median index value (dashed line, PR=1), with 95% confidence bands. Models used the common BMI-free covariate specification; CHNS curves used community-clustered HC0 standard errors, and NHANES curves incorporated WTSAF8YR, SDMVSTRA and SDMVPSU. Corresponding nonlinear Wald tests are reported in Supplementary Table S21. CHNS curves were derived from the n=1,721 complete-case analytic sample, and NHANES curves from the n=2,799 complete-case analytic sample. CHNS, China Health and Nutrition Survey; NHANES, National Health and Nutrition Examination Survey; PR, prevalence ratio; CI, confidence interval.
