# Supplementary material for: Associations of Insulin Resistance Surrogate Indices With Component‐Defined Metabolically Unhealthy Phenotype Among Women With Overweight or Obesity: A Cross‐Sectional Analysis of CHNS and NHANES
Source: Int J Endocrinol. 2026 Oct 14;2026:5660424. doi: 10.1155/ije/5660424 (PMC13662632; doi:10.1155/ije/5660424)
Supplement: Supplementary file 2 [file IJE-2026-5660424-s001.docx]

**STROBE Statement—Checklist of Items to be Included in Reports of Cross-Sectional Studies**

Manuscript: “Associations of insulin resistance surrogate indices with component-defined metabolically unhealthy phenotype among women with overweight or obesity: a cross-sectional analysis of CHNS and NHANES” (Manuscript ID: 5660424)

| **Item** | **Recommendation** | **Reported in manuscript** |
| --- | --- | --- |
| **Title and abstract** | | |
| 1a | (a) Indicate the study's design with a commonly used term in the title or the abstract | Title: “...a cross-sectional analysis of CHNS and NHANES.” |
| 1b | (b) Provide in the abstract an informative and balanced summary of what was done and what was found | Abstract (Background/Methods/Results/Conclusions, structured) |
| **Introduction** | | |
| 2 | Explain the scientific background and rationale for the investigation being reported | Background, paragraphs 1–3 (metabolic heterogeneity in women with overweight/obesity; rationale for surrogate indices) |
| 3 | State specific objectives, including any prespecified hypotheses | Background, final paragraph (“In this study, we evaluated associations of HOMA-IR, TyG, TyG-BMI, TG/HDL-C and METS-IR...”) |
| **Methods** | | |
| 4 | Present key elements of study design early in the paper | Methods—Study design and data sources (first paragraph): cross-sectional analysis, CHNS discovery/NHANES independent replication |
| 5 | Describe the setting, locations, and relevant dates, including periods of recruitment, exposure, follow-up, and data collection | Methods—Study design and data sources: CHNS 2009 biomarker survey; NHANES 2011–2018 combined cycles |
| 6a | Give the eligibility criteria, and the sources and methods of selection of participants | Methods—Study design and data sources: CHNS and NHANES eligibility/restriction criteria; Figure 1 (flowchart) |
| 7 | Clearly define all outcomes, exposures, predictors, potential confounders, and effect modifiers. Give diagnostic criteria, if applicable | Methods—Assessment of surrogate indices (formulas); Definition of metabolically unhealthy phenotype; Covariates; Supplementary Table S1 |
| 8 | For each variable of interest, give sources of data and details of methods of assessment. Describe comparability of assessment methods if more than one group | Methods—Study design and data sources; Assessment of surrogate indices; Supplementary Table S1 (database-specific operational definitions) |
| 9 | Describe any efforts to address potential sources of bias | Methods—Sensitivity and supportive subgroup analyses (formula-to-outcome component-overlap analyses); Discussion—Limitations |
| 10 | Explain how the study size was arrived at | Methods—Study design and data sources (initial and final analytic sample sizes for CHNS and NHANES); Figure 1 |
| 11 | Explain how quantitative variables were handled in the analyses. If applicable, describe which groupings were chosen and why | Methods—Statistical analysis (per-1-SD standardisation; database-specific empirical quartiles, Q1 as reference) |
| 12a | Describe all statistical methods, including those used to control for confounding | Methods—Statistical analysis; Covariates; Composite metabolic dysregulation burden and consistency analysis |
| 12b | Describe any methods used to examine subgroups and interactions | Methods—Sensitivity and supportive subgroup analyses (age-group and BMI-category interaction terms) |
| 12c | Explain how missing data were addressed | Methods—Statistical analysis (“No missing values were imputed... complete cases”) |
| 12d | If applicable, describe analytical methods taking account of sampling strategy | Methods—Statistical analysis (CHNS community-clustered HC0 SEs; NHANES survey-weighted quasipoisson regression with WTSAF8YR/SDMVSTRA/SDMVPSU) |
| 12e | Describe any sensitivity analyses | Methods—Sensitivity and supportive subgroup analyses; Composite metabolic dysregulation burden and consistency analysis |
| **Results** | | |
| 13a–c | Report numbers of individuals at each stage of study; give reasons for non-participation; consider a flow diagram | Results—Participant selection and characteristics; Figure 1 (flowchart) |
| 14a–b | Give characteristics of study participants and information on exposures/potential confounders; indicate missing data | Results—Participant selection and characteristics; Table 1 |
| 15 | Report numbers of outcome events or summary measures | Results—Participant selection and characteristics (unweighted and survey-weighted MUP prevalence) |
| 16a–c | Give unadjusted and confounder-adjusted estimates and their precision; report category boundaries for categorised variables; consider absolute-risk translation if relevant | Results—Main associations and quartile-based gradients; Table 2; Figure 2; Supplementary Table S3 |
| 17 | Report other analyses done—subgroups, interactions, sensitivity analyses | Results—Exploratory internal discrimination; Composite metabolic dysregulation burden and consistency; Sensitivity and supportive subgroup analyses; Supplementary Materials |
| **Discussion** | | |
| 18 | Summarise key results with reference to study objectives | Discussion, first paragraph |
| 19 | Discuss limitations, taking into account sources of potential bias or imprecision; discuss direction and magnitude of bias | Discussion—Strengths and limitations (Limitations subsection) |
| 20 | Give a cautious overall interpretation considering objectives, limitations, multiplicity of analyses, and other evidence | Discussion, throughout; Conclusions |
| 21 | Discuss the generalisability (external validity) of the study results | Discussion—population-level characterisation paragraph (cross-database consistency sentence); Limitations (final sentence); Conclusions |
| **Other information** | | |
| 22 | Give the source of funding and the role of the funders for the present study | Declarations—Funding |

Note. This checklist is based on the STROBE Statement checklist for cross-sectional studies (von Elm E, Altman DG, Egger M, et al. Ann Intern Med. 2007;147(8):573-577; https://www.strobe-statement.org). “Reported in manuscript” locations refer to section and paragraph headings in the revised manuscript, consistent with the version submitted for this revision.
